# How directed evolution reshapes energy landscapes in enzymes to boost catalysis

**DOI:** 10.1126/science.abd3623

**Published:** 2020-11-19

**Authors:** Renee Otten, Ricardo A. P. Pádua, H. Adrian Bunzel, Vy Nguyen, Warintra Pitsawong, MacKenzie Patterson, Shuo Sui, Sarah L. Perry, Aina E. Cohen, Donald Hilvert, Dorothee Kern

**Affiliations:** 1 Howard Hughes Medical Institute and Department of Biochemistry, Brandeis University, 415 South Street, Waltham, MA 02454, USA; 2 Laboratory of Organic Chemistry, ETH Zurich, 8093 Zurich, Switzerland; 3 Department of Chemical Engineering, Institute of Applied Life Sciences, University of Massachusetts Amherst, Amherst, MA 01003, USA; 4 Stanford Synchrotron Radiation Lightsource, 2575 Sand Hill Road, Menlo Park, CA 94025, USA

## Abstract

The advent of biocatalysts designed computationally and optimized by laboratory evolution provides an opportunity to explore molecular strategies for augmenting catalytic function. Applying a suite of NMR, crystallographic, and stopped-flow techniques to an enzyme designed for an elementary proton transfer reaction, we show how directed evolution gradually altered the conformational ensemble of the protein scaffold to populate a narrow, highly active conformational ensemble and achieve a nearly billionfold rate acceleration. Mutations acquired during optimization enabled global conformational changes, including high-energy backbone rearrangements, that cooperatively organized the catalytic base and oxyanion stabilizer, thus perfecting transition-state stabilization. Explicit sampling of conformational sub-states during design, and specifically stabilizing productive over all unproductive conformations, could speed up the development of protein catalysts for many chemical transformations.

## One Sentence Summary

Altered sampling of conformational sub-states on multiple time scales was critical for optimization of a designer enzyme.

Computational enzyme design has afforded catalysts for chemical reactions ranging from ester hydrolysis to abiological cycloadditions (1, 2). Although starting activities are usually low, they can be increased to levels approaching those of natural enzymes through laboratory evolution (3-6). This process mimics the natural selection of enzymes in biology, with the advantage that individual intermediates along the evolutionary pathway can be characterized to deduce how function was enhanced. A comprehensive understanding of the molecular changes that confer better activity could improve design protocols as well as guide the development of smarter mutagenesis and screening strategies.

**Fig. 1.**
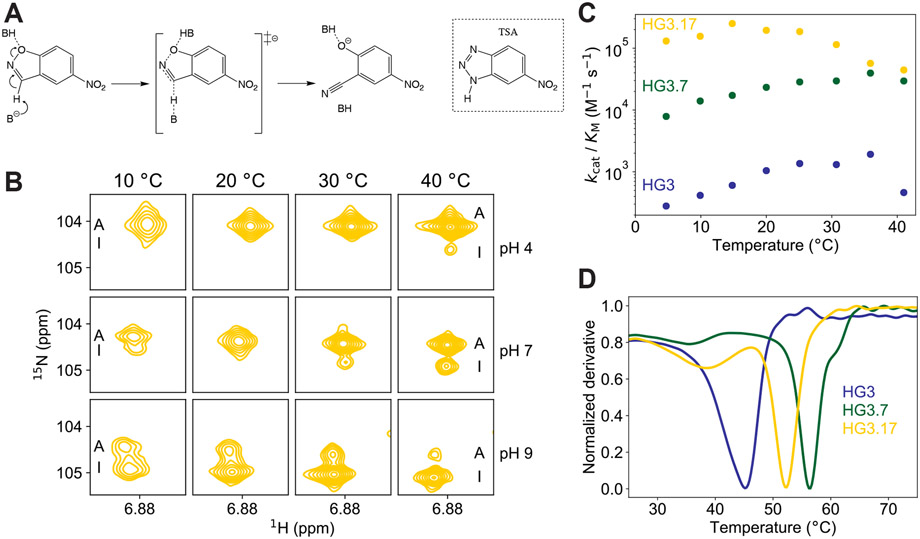
Inactivation of Kemp eliminase variants is due to sampling of an alternative, folded conformation. **(A)** The Kemp elimination reaction (8) with the structure of the transition-state analog shown on the right. **(B)** Temperature- and pH-dependent NMR experiments for free HG3.17 display peak duplication for many residues (Fig. S2A) as exemplified here for Gly263. The cross peak of the minor, inactive (I) species increases with temperature and/or pH, indicative of a slow interconversion process between two folded conformations. **(C)** Directed evolution greatly increased catalytic efficiency (*k*_cat_/*K*_M_) from HG3 to HG3.17 (3), but for evolved enzymes a clear temperature-dependent inactivation is observed. **(D)** Protein stability measurements using thermal-shift assays indicate that inactivation above ~25 °C is not due to global unfolding, but the smaller transition at lower temperatures suggests the presence of another state.

Here, we investigate the molecular origins of the nearly billionfold rate enhancement achieved by directed evolution of the computationally designed Kemp eliminase HG3 (7). The Kemp elimination (Fig. 1A) is a well-studied model for proton transfer from carbon (8) that has served as a benchmark for *de novo* design (7, 9-11). Although the first-generation HG3 design is significantly more efficient than an “off-the-shelf” catalyst like bovine serum albumin (12), its specific activity was further increased 200-fold over 17 rounds of mutagenesis and screening (3). The resulting catalyst HG3.17, which exhibits improved alignment of the substrate and the catalytic base (Asp127) and possesses a newly acquired H-bond donor for oxyanion stabilization (Gln50), approaches the efficiency of natural enzymes that promote metabolically important proton transfers (13). Characterization of HG3, the evolutionary intermediate HG3.7, and optimized HG3.17 by a combination of NMR spectroscopy, cryo- and high-temperature crystallography, and stopped-flow fluorescence experiments shows that altered sampling of conformational sub-states on different temporal and spatial scales was crucial for attaining the evolved enzyme’s superior catalytic power.

We first obtained NMR backbone assignments for HG3.17 (Fig. S1) and recorded data at different temperatures and pH values (Fig. 1B and Fig. S2). Unexpectedly, peak duplication spanning a large portion of the protein was detected (Fig. S2A), indicating that the resting enzyme exists in two different folded states undergoing a global conformational exchange that is slow on the NMR timescale. Upon raising either temperature or pH, the cross-peak intensity of one set of peaks increased relative to the other (Fig. 1B and Fig. S2B-D). Based on the independent observation that HG3.17 undergoes inactivation above ~25 °C (Fig. 1C), well below the melting temperature (*T*_m_ ≥ 50 °C; Fig. 1D), we hypothesized that the species observed at high temperature corresponds to a less active (or fully inactive) conformational sub-state. An additional transition observed in thermal-shift assays (Fig. 1D and Fig. S3), not seen in circular dichroism melting curves (3), provides further evidence for a pre-existing equilibrium between active (A) and inactive (I) forms of the enzyme.

**Fig. 2.**
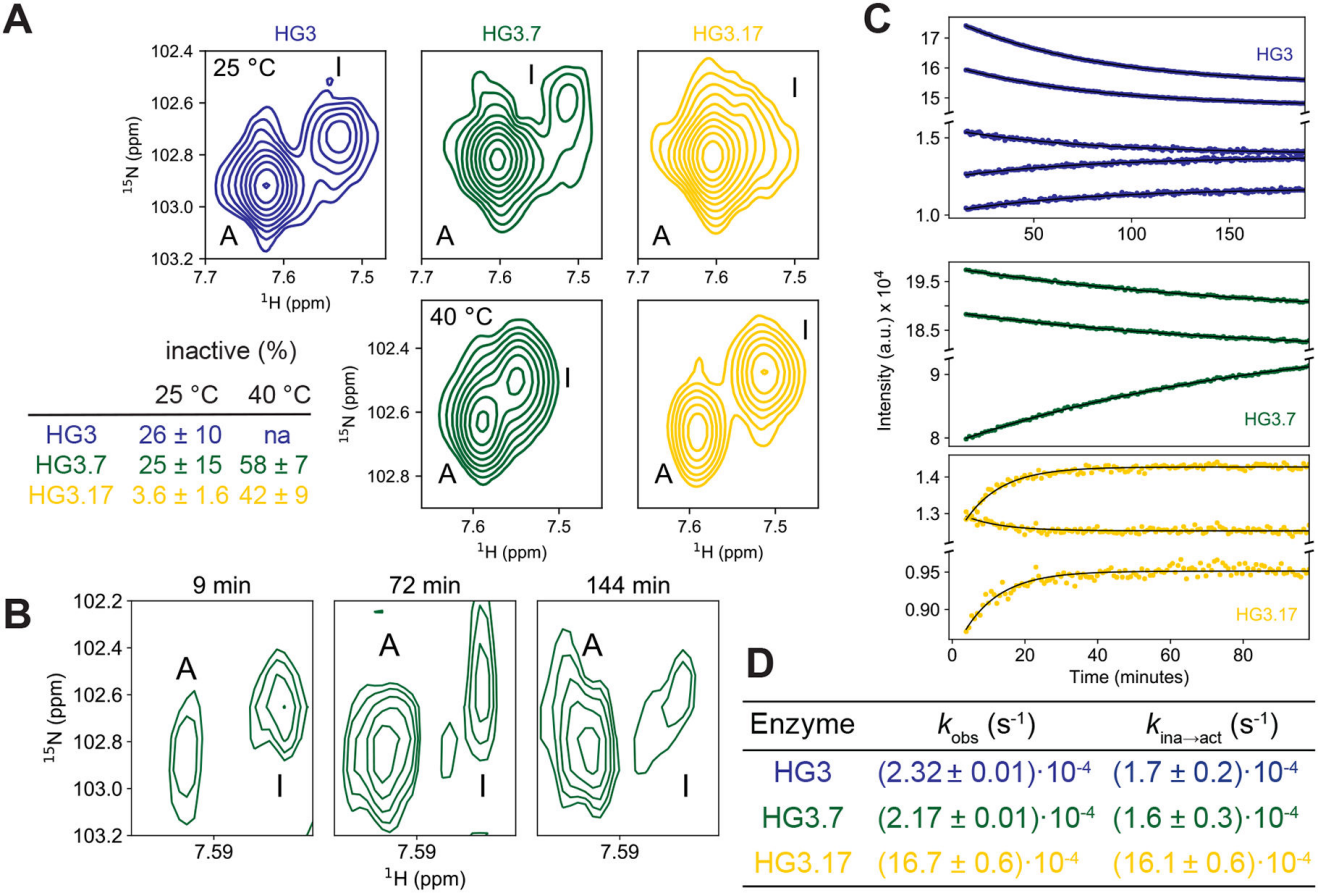
Characterization of the inactive/active interconversion of Kemp eliminase variants. **(A)** Active and inactive conformations are observed for all HG3 variants as exemplified by the NMR cross peaks of Gly229 at pH 7. At 25 °C the inactive population is small for HG3.17, but sizeable for HG3 and HG3.7, and the inactive species increases with temperature. **(B-D)** Detection of interconversion kinetics at 25 °C by real-time NMR using a pH-jump from proteins equilibrated at pH 10.0 to 7.0. **(B)** pH-jump experiment for HG3.7 followed by 2D HSQC spectra confirm that the interconversion from the inactive (at high pH) to active (at lower pH) conformation indeed occurs, but the quality of the data is insufficient to extract reliable rate constants. **(C, D)** The measurements were repeated using 1D proton NMR experiments and time-dependent changes of selected peak areas are shown **(C).** Observed rate constants (*k*_obs_) in **(C),** combined with the populations from NMR **(A)**, yielded the activation rate constant (*k*_ina→act_; **D).**

Importantly, the NMR spectra of HG3 and HG3.7 show analogous features, although their inactive sub-states are populated to a greater extent than in HG3.17 (Fig. S2C,D). Estimating the respective populations from the volumes of duplicated cross peaks (Fig. 2A) shows that the inactive species comprises ~25% of the HG3 and HG3.7 samples at 25 °C, but only 5% of HG3.17. At 40 °C, though, the fraction of inactive state increases to 42% and 58% for HG3.17 and HG3.7, respectively (Fig. 2A). Activity-based pH-jump assays (Fig. S4) confirmed that the inactivation process is fully reversible, and repopulation of the active species could be monitored in real time by recording two-dimensional NMR spectra after a rapid change from pH 10 to 7 (Fig. 2B). Trp fluorescence (Fig. S5) and one-dimensional NMR (Fig. 2C) experiments allowed extraction of quantitative rate constants (*k*_ina→act_; Fig. 2D) and show that the interconversion between the two states is slow for all variants (*k*_obs_ ~10^−3^-10^−4^ s^−1^). We note that in addition to this slow process, millisecond motions are detected for many residues in the form of line-broadening or complete loss of amide signals for several residues in the core β-strands (Fig. S1D). We hypothesize that these faster, more localized motions underlie the slower collective global rearrangements we observe.

Taken together, these data show that: (i) the HG3 variants all exhibit a slow, pre-existing equilibrium between active and inactive conformational sub-states; (ii) the last 10 rounds of directed evolution (HG3.7→G3.17) substantially reduced the population of inactive species present under ambient conditions; and (iii) moving away from the conditions employed for selection (i.e., higher temperatures or pH) increases the fraction of enzyme in the inactive state.

**Fig. 3.**
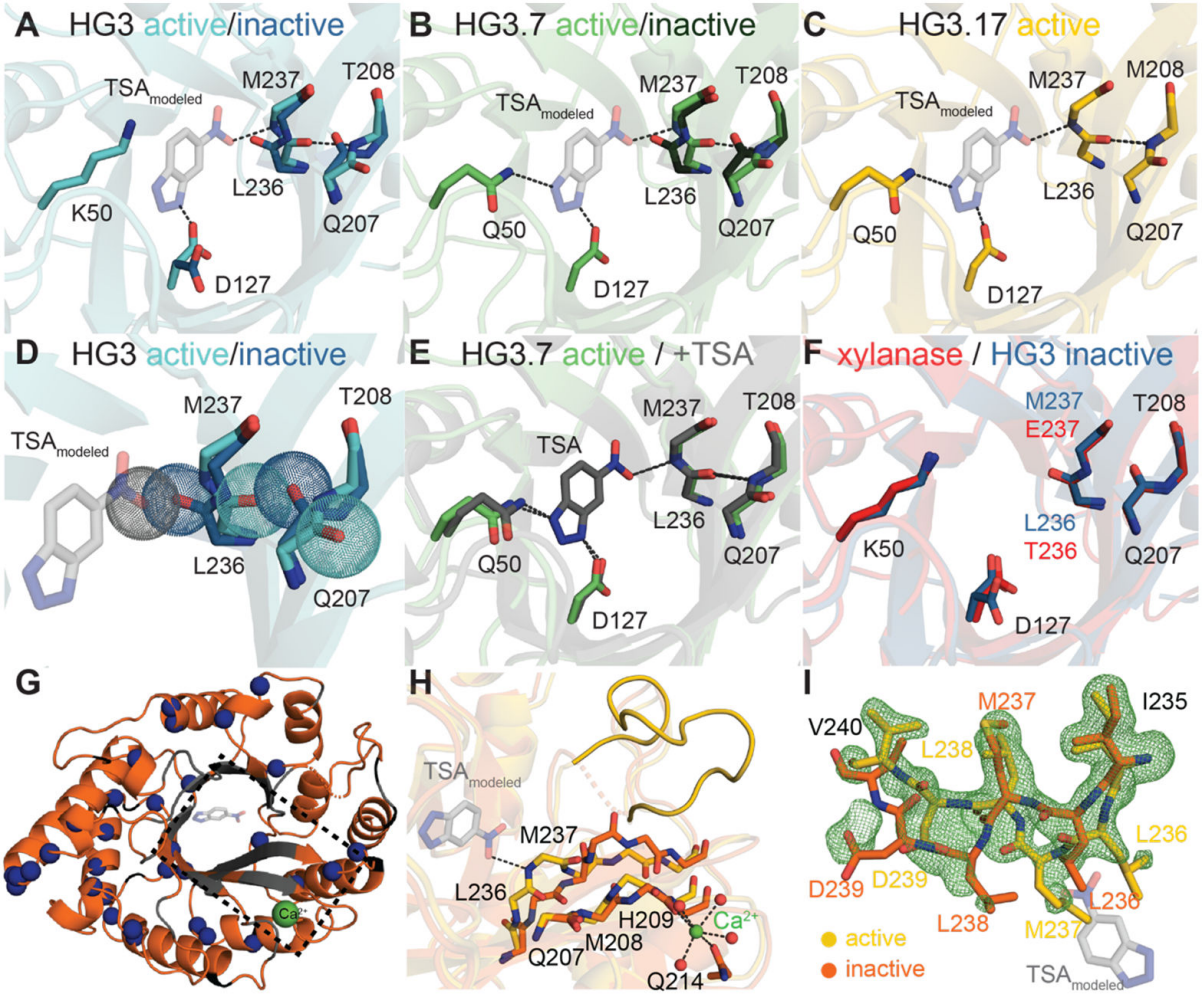
X-ray data reveal extensive structural changes between the active and inactive conformations of the Kemp eliminases. **(A-E)** Crystal structures in the absence of TSA show two conformations for residues near the active site of HG3 **(A)** and HG3.7 **(B)**, but not of HG3.17 **(C)**. The active state (light colors) makes favorable interactions with the modeled TSA (transparent gray; **A-C**), whereas the inactive state (dark colors) is a binding-incompetent conformation as the carbonyl-group of Leu236 would clash with the TSA **(D)**. **(E)** The active conformation of free HG3.7 is nearly superimposable with its TSA-bound form. **(F)** The inactive backbone conformation is the only one observed in the xylanase scaffold (red, PDB 1gor (14)). **(G)** X-ray structure of inactive conformation of HG3.17, obtained by calcium (green) binding at a surface-exposed site. Residues with NMR peak duplication (Fig. 1B) are shown in blue, unassigned residues in grey, and prolines in black. **(H)** Superposition of the active (yellow) and inactive, calcium-bound (orange) conformation of HG3.17 shows the propagation of backbone changes from the calcium-binding site extending to the active site with modeled TSA. **(I)** The mFo-DFc-polder map (green mesh, contoured at 3σ) for crystallographic data recorded at 70 °C for free HG3.17 can only be explained by modeling both the active (yellow) and inactive (orange) conformations (see also Fig. S7H).

To provide structural information on these conformational sub-states, we turned to X-ray crystallography. Cryogenic structures of HG3 and HG3.7 in the absence of a ligand revealed that β-strands 6 and 7 (located adjacent to the binding pocket), adopt two distinct backbone conformations whereas only a single conformer is observed for HG3.17 (Fig. 3A-C, Fig. S6, and Table S1). One conformation matches that seen in the corresponding complexes with a transition-state analog (TSA), and presumably represents the active sub-state. The almost identical positioning of the catalytic residues in the free and the TSA-bound forms of HG3.7 (Fig. 3E and Table S1) indicates that the active site of this sub-state is already primed for catalysis. In the inactive sub-state, however, a backbone flip creates a steric clash between the carbonyl group of Leu236 and the nitro group of the TSA that would block ligand binding (Fig. 3D). Interestingly, this inactive backbone conformation is the only one present in the original xylanase scaffold used for design (PDB 1gor (14); Fig. 3F).

Because the sparsely populated inactive sub-state of HG3.17 was not detected in the cryogenic X-ray structure, we set up crystal screens under conditions favoring this conformation (pH 10 and 37 °C). Crystals obtained with calcium in the crystallization solution yielded a structure of this inactive species (Fig. 3G,H and Table S1). A weak, surface-exposed calcium-binding site stabilizes the inactive form, with substantial backbone changes propagating to the active site, including the backbone flip in strand 7 that impedes substrate binding (Fig. 3G,H and Fig. S7A-E). The 270-282 loop, which contains four of the 10 mutations introduced in the last rounds of directed evolution, also differs in the two conformational sub-states. This segment is ordered in the active state, likely stabilized by a cation-π interaction between protonated His209 and Phe276 (Fig. 3H and Fig. S8A), but disordered in the inactive state. We conjecture that disrupting the His209-Phe276 interaction by raising either temperature or pH, or mutation of His to Ala (Fig. S8B,C), shifts the equilibrium toward the inactive conformation. NMR spectra of HG3.17 with 100 mM Ca^2+^ confirm that the conformational sub-state captured crystallographically is the same as the inactive species in solution, as the positions of the corresponding cross peaks are virtually unaltered but their intensities relative to the ‘active’ signal increase (Fig. S7F,G). Independent kinetic measurements show that the enzyme is 80% inhibited in the crystallization buffer. Remarkably, a HG3.17 structure obtained at 70 °C in the absence of calcium enabled simultaneous observation of both the inactive and active conformations (Fig. 3I, Fig. S7H, and Table S1) as observed in solution by NMR. At this temperature, the complete global rearrangement is permitted within the crystal lattice.

**Fig. 4.**
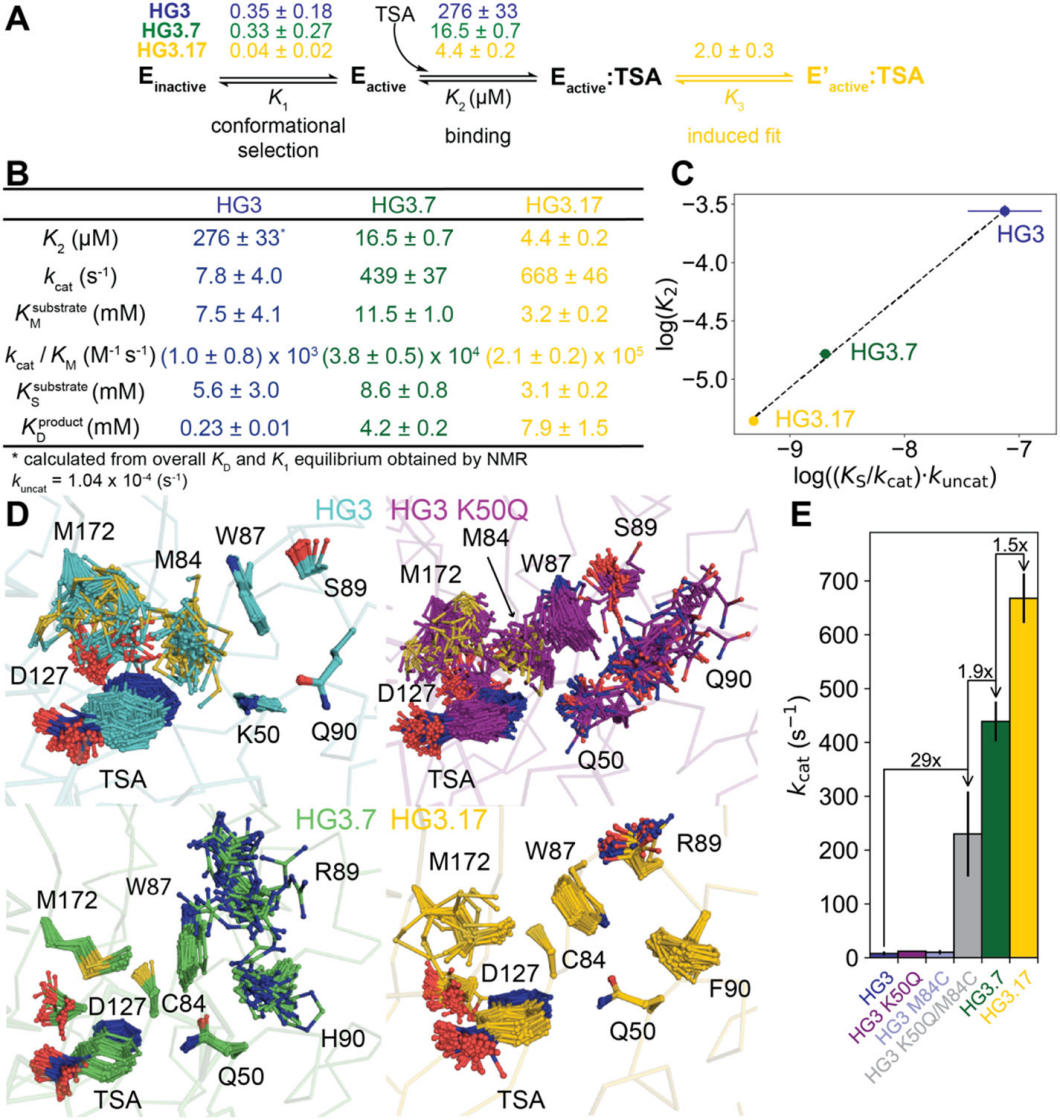
Transition-state analogue binding as a proxy for probing the chemical activation barrier over evolution. **(A)** Mechanism and microscopic equilibrium constants (reported as dissociation constants) for TSA binding to the HG3 variants. **(B)** Kinetic parameters obtained by numerically fitting the progress curves for substrate conversion at 25 °C to an extended Michaelis-Menten model (Fig. S14A). **(C)** The increase in (*K*_S_/*k*_cat_)·*k*_uncat_ through the evolutionary rounds correlates remarkably well with the change in *K*_2_, as expected from transition-state theory. **(D)** Ensemble refinements (see also Fig. S16-S17) of cryogenic X-ray structures of HG3 variants bound to TSA reveal extensive conformational sampling for HG3 and HG3 K50Q, whereas in evolved enzymes the side chain orientations become more ordered leading to optimal positioning of the catalytic base Asp127 and the oxyanion stabilizer Gln50 (see also Fig. S15-S16). The apparent order for residues Lys50, Trp87, Ser89, and Gln90 in HG3 is explained by crystal contacts in that region (Fig. S16D) that are specific to HG3. HG3 K50Q is thus better suited for comparison of the ensembles as it forms similar crystal contacts as HG3.7 and HG3.17. **(E)** *k*_cat_ values for all Kemp eliminase variants (Fig. S18) highlight the major boost in *k*_cat_ by the K50Q/M84C substitutions.

Considering that an enzyme’s affinity to an ideal transition-state analogue is directly proportional to the rate enhancement for the chemical step (15, 16), we dissected the TSA binding mechanism to probe changes in the activation barrier of the chemical step through directed evolution. The minimal binding scheme involves a conformational-selection step for the binding-competent state plus the physical binding step, and—for HG3.17 only—an additional induced-fit step (Fig. 4A and Fig. S9). We hypothesize that the induced-fit step involves a slow ring flip of Trp44 at the bottom of the binding pocket (Fig. S9E), but this does not likely affect activity significantly. The microscopic rate constants were obtained by combining stopped-flow binding kinetics and NMR experiments (Figs. 2 and 4A and Figs. S10-S13); the agreement between the measured macroscopic *K*_D_ and their calculated values (Fig. S13E and Equations 11 and 12) confirm our binding schemes. As expected for a good TSA, its affinity increases over the course of evolution (*K*_2_ values of 276, 16.5, and 4.4 μM). For a quantitative comparison of these values with improvements in catalytic efficiency, reliable steady-state parameters are paramount. Previously, *k*_cat_ and *K*_M_ values were extracted from initial rates, but our new insights into these enzymes reveal that a simple Michaelis-Menten model is not sufficient to describe the system. We therefore monitored the enzymatic conversion of 5-nitrobenzisoxazole to completion and numerically fit the data to a scheme that includes the conformational-selection step and product inhibition (Fig. S14A). This approach enables a more reliable determination of *k*_cat_ and *K*_M_ even if substrate saturation cannot be achieved (17), as is the case for 5-nitrobenzoisoxazole due to its limited solubility (Fig. 4B and Fig. S14). The extracted values show excellent agreement with the previously published steady-state parameters after correction for the fraction of enzyme in the active state, and the increase in (*K*_S_/*k*_cat_)·*k*_*un*cat_ through the evolutionary rounds indeed correlates remarkably well with the change in *K*_2_ (Fig. 4C). Notably, as TSA affinity increased during evolution, product affinity decreased, minimizing product inhibition and guaranteeing efficient enzyme turnover (Fig. 4B).

Although reducing the fraction of inactive sub-states in the apo protein improved overall catalysis, the maximum change in the active population of 20% between variants only accounts for a small fraction of the observed 200-fold increase in catalytic efficiency from HG3 to HG3.17 (Fig. 1C). However, ensemble refinement of crystal structures (18) for all the variants in complex with the TSA points to progressive increase of the active configuration as the key contributor to the catalytic enhancement (Figs. 3A-F and 4D and Figs. S15-S17). Increased ordering of the Met172 and Met237 side chains, which interact with one face of the TSA and likely stabilize the charge delocalized transition state through London dispersion forces (19-21), illustrates this trend (Fig. 4D and Figs. S15E,F and S16C). Although relatively flexible in HG3, Met172 becomes better ordered in HG3.7 due to shortening of residue 84 through the M84C mutation, which enabled a stabilizing interaction between the terminal methyl group of Met172 and the π-face of Trp87. The resulting conformation helps to position the catalytic base (Asp127), which samples many unreactive conformations in HG3, in a single orientation with the geometry required for proton abstraction (Figs. 3A-F and 4D and Figs. S15 and S16). Furthermore, HG3.7’s acquisition of the oxyanion stabilizer Gln50 constrains the TSA in a productive pose through hydrogen bonding, which is accompanied by ordering of Met237. In principle, Lys50 in HG3 could act as an effective oxyanion stabilizer and constrain the ligand in a reactive pose, but its side chain points away and forms a hydrogen bond with Gln90 instead (Fig. S15A,B). Further tuning of active site conformations by second- and third-shell mutations from subsequent evolutionary rounds ultimately yielded the highly preorganized HG3.17 binding pocket (Fig. 4D and Figs. S15 and S16).

To disentangle the catalytic contributions of M84C and K50Q from those of more distant mutations, we introduced them singly and together into the original computational design. K50Q increased HG3 activity only 1.5-fold (Fig. 4E and Fig. S18), in marked contrast to the 40-fold loss in efficiency seen when Gln50 was reverted to Lys in HG3.17 (22). The maximum likelihood X-ray structure of HG3 K50Q shows that the Gln50 side chain is properly oriented to hydrogen-bond with the TSA (Fig. S15E), but it shows significant disorder in the ensemble refinement, as do Asp127 and adjacent residues (Fig. 4D and Fig. S16). Similarly, M84C provides no catalytic benefit to HG3 on its own. Together, however, these two mutations boost catalytic efficiency substantially, increasing the rate of the chemical step ~30-fold and overall catalytic efficiency 10-fold (Fig. 4E and Fig. S18). Epistasis is indicated by the synergistic effect of these two mutations. This striking result highlights the serendipitous paths that directed evolution takes, and offers a bright outlook for rational enzyme design: only two of the 17 mutations introduced by directed evolution (Fig. S19) account for a major fraction of the catalytic enhancement. Crucially, these two mutations were predicted from a structural analysis within a protein dynamics framework, underscoring the potential for improved success in enzyme design by focusing on counterselection against sampling of alternative, catalytically unproductive states. The other 15 mutations in HG3.17 had a relatively small effect on *k*_cat_ (3-fold), but increased *k*_cat_/*K*_M_ 10-fold by lowering *K*_M_; they also largely eliminated the inactive conformational sub-state and decreased product inhibition (Fig. 4B).

Kemp eliminase HG3.17 is among the most efficient artificial enzymes described to date. Analysis of its evolutionary trajectory has revealed how changes in conformational sampling were critical to its success. All HG3 variants have an inactive conformational sub-state, rooted in the original protein scaffold, which was gradually supplanted with a catalytically competent sub-state as evolution progressed. Although conformational selection has been observed in the optimization of other designed enzymes (23, 24), including Kemp eliminases (4, 25-28), what is striking in the HG3 system is that the active conformational sub-state was not explicitly engineered into the xylanase scaffold but only emerged upon introduction of the 11 design mutations due to a serendipitous backbone flip. Instead, design created two “energetically frustrated” enzyme conformations. Drastically decreased sampling of unreactive conformations within the catalytically-competent state provided the second major mechanism for improving efficiency. While distant mutations contributed to this fine-tuning, two active-site residues played an outsized role in sculpting a steric and electrostatic environment conducive to transition-state stabilization. These findings speak to the ongoing debate on the role of protein dynamics in enzyme catalysis (29-34), providing a direct, quantitative demonstration of how modulating a protein conformational landscape, something not optimized by current design protocols but which evolution perfects, can speed up a simple chemical reaction. Proper modeling of conformational dynamics and selective stabilization of productive sub-states over all unproductive conformations during design, for example by explicit energy landscape optimization (35), may open the door to substantially better biocatalysts.

## Supplementary Material

1

## References

[1] Kiss G, Celebi-Olcum N, Moretti R, Baker D, Houk KN, Computational enzyme design. Angew Chem Int Ed Engl 52, 5700–5725 (2013). 23526810 10.1002/anie.201204077

[2] Kries H, Blomberg R, Hilvert D, De novo enzymes by computational design. Curr Opin Chem Biol 17, 221–228 (2013). 23498973 10.1016/j.cbpa.2013.02.012

[3] Blomberg R , Precision is essential for efficient catalysis in an evolved Kemp eliminase. Nature 503, 418–421 (2013). 24132235 10.1038/nature12623

[4] Khersonsky O , Bridging the gaps in design methodologies by evolutionary optimization of the stability and proficiency of designed Kemp eliminase KE59. Proc Natl Acad Sci U S A 109, 10358–10363 (2012). 22685214 10.1073/pnas.1121063109 PMC3387081

[5] Obexer R , Emergence of a catalytic tetrad during evolution of a highly active artificial aldolase. Nat Chem 9, 50–56 (2017). 27995916 10.1038/nchem.2596

[6] Preiswerk N , Impact of scaffold rigidity on the design and evolution of an artificial Diels-Alderase. Proc Natl Acad Sci U S A 111, 8013–8018 (2014). 24847076 10.1073/pnas.1401073111 PMC4050586

[7] Privett HK , Iterative approach to computational enzyme design. Proc Natl Acad Sci U S A 109, 3790–3795 (2012). 22357762 10.1073/pnas.1118082108 PMC3309769

[8] Casey ML, Kemp DS, Paul KG, Cox DD, Physical organic chemistry of benzisoxazoles. I. Mechanism of the base-catalyzed decomposition of benzisoxazoles. J Org Chem 38, 2294–2301 (1973).

[9] Thorn SN, Daniels RG, Auditor MT, Hilvert D, Large rate accelerations in antibody catalysis by strategic use of haptenic charge. Nature 373, 228–230 (1995). 7816136 10.1038/373228a0

[10] Rothlisberger D , Kemp elimination catalysts by computational enzyme design. Nature 453, 190–195 (2008). 18354394 10.1038/nature06879

[11] Korendovych IV , Design of a switchable eliminase. Proc Natl Acad Sci U S A 108, 6823–6827 (2011). 21482808 10.1073/pnas.1018191108 PMC3084051

[12] Hollfelder F, Kirby AJ, Tawfik DS, Off-the-shelf proteins that rival tailor-made antibodies as catalysts. Nature 383, 60–62 (1996). 8779715 10.1038/383060a0

[13] Knowles JR, Enzyme catalysis: not different, just better. Nature 350, 121–124 (1991). 2005961 10.1038/350121a0

[14] Lo Leggio L , Substrate specificity and subsite mobility in T. aurantiacus xylanase 10A. FEBS Lett 509, 303–308 (2001). 11741607 10.1016/s0014-5793(01)03177-5

[15] Mader MM, Bartlett PA, Binding Energy and Catalysis: The Implications for Transition-State Analogs and Catalytic Antibodies. Chem Rev 97, 1281–1302 (1997). 11851452 10.1021/cr960435y

[16] Westerik JOC, Wolfenden R, Aldehydes as Inhibitors of Papain. J Biol Chem 247, 8195–8197 (1972). 4640942

[17] Johnson KA, Kinetic Analysis for the New Enzymology. (KinTek Coprporation, Austin, TX, USA, ed. 1, 2019).

[18] Burnley BT, Afonine PV, Adams PD, Gros P, Modelling dynamics in protein crystal structures by ensemble refinement. Elife 1, e00311 (2012). 23251785 10.7554/eLife.00311 PMC3524795

[19] Kemp DS, Cox DD, Paul KG, Physical organic chemistry of benzisoxazoles. IV. Origins and catalytic nature of the solvent rate acceleration for the decarboxylation of 3-carboxybenzisoxazoles. J Am Chem Soc 97, 7312–7318 (1975).

[20] Valley CC , The methionine-aromatic motif plays a unique role in stabilizing protein structure. J Biol Chem 287, 34979–34991 (2012). 22859300 10.1074/jbc.M112.374504 PMC3471747

[21] Pollice R, Chen P, A Universal Quantitative Descriptor of the Dispersion Interaction Potential. Angew Chem Int Ed Engl 58, 9758–9769 (2019). 31106508 10.1002/anie.201905439

[22] Kries H, Bloch JS, Bunzel HA, Pinkas DM, Hilvert D, Contribution of Oxyanion Stabilization to Kemp Eliminase Efficiency. ACS Catalysis 10, 4460–4464 (2020).

[23] Campbell EC , Laboratory evolution of protein conformational dynamics. Curr Opin Struct Biol 50, 49–57 (2018). 29120734 10.1016/j.sbi.2017.09.005

[24] Maria-Solano MA, Serrano-Hervas E, Romero-Rivera A, Iglesias-Fernandez J, Osuna S, Role of conformational dynamics in the evolution of novel enzyme function. Chem Commun (Camb) 54, 6622–6634 (2018). 29780987 10.1039/c8cc02426j PMC6009289

[25] Khersonsky O , Evolutionary optimization of computationally designed enzymes: Kemp eliminases of the KE07 series. J Mol Biol 396, 1025–1042 (2010). 20036254 10.1016/j.jmb.2009.12.031

[26] Khersonsky O , Optimization of the in-silico-designed kemp eliminase KE70 by computational design and directed evolution. J Mol Biol 407, 391–412 (2011). 21277311 10.1016/j.jmb.2011.01.041 PMC3889864

[27] Hong NS , The evolution of multiple active site configurations in a designed enzyme. Nat Commun 9, 3900 (2018). 30254369 10.1038/s41467-018-06305-y PMC6156567

[28] Broom A , Evolution of an enzyme conformational ensemble guides design of an efficient biocatalyst. bioRxiv doi: 10.1101/2020.03.19.999235 (2020).

[29] Kohen A, Role of dynamics in enzyme catalysis: substantial versus semantic controversies. Acc Chem Res 48, 466–473 (2015). 25539442 10.1021/ar500322s PMC4334245

[30] Hammes-Schiffer S, Benkovic SJ, Relating protein motion to catalysis. Annu Rev Biochem 75, 519–541 (2006). 16756501 10.1146/annurev.biochem.75.103004.142800

[31] Nashine VC, Hammes-Schiffer S, Benkovic SJ, Coupled motions in enzyme catalysis. Curr Opin Chem Biol 14, 644–651 (2010). 20729130 10.1016/j.cbpa.2010.07.020 PMC2953590

[32] Nagel ZD, Klinman JP, A 21st century revisionist's view at a turning point in enzymology. Nat Chem Biol 5, 543–550 (2009). 19620995 10.1038/nchembio.204

[33] Warshel A , Electrostatic basis for enzyme catalysis. Chem Rev 106, 3210–3235 (2006). 16895325 10.1021/cr0503106

[34] Boehr DD, Dyson HJ, Wright PE, An NMR perspective on enzyme dynamics. Chem Rev 106, 3055–3079 (2006). 16895318 10.1021/cr050312q

[35] Norn C , Protein sequence design by explicit energy landscape optimization. bioRxiv doi: 10.1101/2020.07.23.218917, (2020).

[36] Oliphant TE, A guide to NumPy. (Trelgol Publishing, USA, ed. 1, 2006).

[37] Hunter JD, Matplotlib: A 2D Graphics Environment. Computing in Science & Engineering 9, 90–95 (2007).

[38] Battye TG, Kontogiannis L, Johnson O, Powell HR, Leslie AG, iMOSFLM: a new graphical interface for diffraction-image processing with MOSFLM. Acta Crystallogr D Biol Crystallogr 67, 271–281 (2011). 21460445 10.1107/S0907444910048675 PMC3069742

[39] Kabsch W, Xds. Acta Crystallogr D Biol Crystallogr 66, 125–132 (2010). 20124692 10.1107/S0907444909047337 PMC2815665

[40] Evans PR, Murshudov GN, How good are my data and what is the resolution? Acta Crystallogr D Biol Crystallogr 69, 1204–1214 (2013). 23793146 10.1107/S0907444913000061 PMC3689523

[41] Liebschner D , Macromolecular structure determination using X-rays, neutrons and electrons: recent developments in Phenix. Acta Crystallogr D Struct Biol 75, 861–877 (2019). 31588918 10.1107/S2059798319011471 PMC6778852

[42] McCoy AJ , Phaser crystallographic software. J Appl Crystallogr 40, 658–674 (2007). 19461840 10.1107/S0021889807021206 PMC2483472

[43] Afonine PV , Towards automated crystallographic structure refinement with phenix.refine. Acta Crystallogr D Biol Crystallogr 68, 352–367 (2012). 22505256 10.1107/S0907444912001308 PMC3322595

[44] Emsley P, Lohkamp B, Scott WG, Cowtan K, Features and development of Coot. Acta Crystallogr D Biol Crystallogr 66, 486–501 (2010). 20383002 10.1107/S0907444910007493 PMC2852313

[45] Kowiel M, Jaskolski M, Dauter Z, ACHESYM: an algorithm and server for standardized placement of macromolecular models in the unit cell. Acta Crystallogr D Biol Crystallogr 70, 3290–3298 (2014). 25478846 10.1107/S1399004714024572 PMC4257622

[46] The PyMOL Molecular Graphics System, version 2.4. (Schrödinger, LLC, New York, 2020).

[47] Touw WG , A series of PDB-related databanks for everyday needs. Nucleic Acids Res 43, D364–368 (2015). 25352545 10.1093/nar/gku1028 PMC4383885

[48] Kabsch W, Sander C, Dictionary of protein secondary structure: pattern recognition of hydrogen-bonded and geometrical features. Biopolymers 22, 2577–2637 (1983). 6667333 10.1002/bip.360221211

[49] Theobald DL, Steindel PA, Optimal simultaneous superpositioning of multiple structures with missing data. Bioinformatics 28, 1972–1979 (2012). 22543369 10.1093/bioinformatics/bts243 PMC3400950

[50] Jubb HC , Arpeggio: A Web Server for Calculating and Visualising Interatomic Interactions in Protein Structures. J Mol Biol 429, 365–371 (2017). 27964945 10.1016/j.jmb.2016.12.004 PMC5282402

[51] Tickle IJ, Statistical quality indicators for electron-density maps. Acta Crystallogr D Biol Crystallogr 68, 454–467 (2012). 22505266 10.1107/S0907444911035918 PMC3322605

[52] Zheng H , CheckMyMetal: a macromolecular metal-binding validation tool. Acta Crystallogr D Struct Biol 73, 223–233 (2017). 28291757 10.1107/S2059798317001061 PMC5349434

[53] Delaglio F , NMRPipe: a multidimensional spectral processing system based on UNIX pipes. J Biomol NMR 6, 277–293 (1995). 8520220 10.1007/BF00197809

[54] Lee W, Tonelli M, Markley JL, NMRFAM-SPARKY: enhanced software for biomolecular NMR spectroscopy. Bioinformatics 31, 1325–1327 (2015). 25505092 10.1093/bioinformatics/btu830 PMC4393527

[55] Niklasson M , Comprehensive analysis of NMR data using advanced line shape fitting. J Biomol NMR 69, 93–99 (2017). 29043470 10.1007/s10858-017-0141-6 PMC5662661

[56] Zhu G, Bax A, Improved Linear Prediction for Truncated Signals of Known Phase. J Magn Reson 90, 405–410 (1990).

[57] Zhu G, Bax A, Two-Dimensional Linear Prediction for Signals Truncated in Both Dimensions. J Magn Reson 98, 192–199 (1992).

[58] Muhandiram DR, Kay LE, Gradient-Enhanced Triple-Resonance Three-Dimensional NMR Experiments with Improved Sensitivity. J Magn Reson, Ser B 103, 203–216 (1994).

[59] Zhang O, Kay LE, Olivier JP, Forman-Kay JD, Backbone 1H and 15N resonance assignments of the N-terminal SH3 domain of drk in folded and unfolded states using enhanced-sensitivity pulsed field gradient NMR techniques. J Biomol NMR 4, 845–858 (1994). 7812156 10.1007/BF00398413

[60] Weigelt J, Single Scan, Sensitivity- and Gradient-Enhanced TROSY for Multidimensional NMR Experiments. J Am Chem Soc 120, 10778–10779 (1998).

[61] Kay L, Keifer P, Saarinen T, Pure absorption gradient enhanced heteronuclear single quantum correlation spectroscopy with improved sensitivity. J Am Chem Soc 114, 10663–10665 (1992).

[62] Tugarinov V, Kanelis V, Kay LE, Isotope labeling strategies for the study of high-molecular-weight proteins by solution NMR spectroscopy. Nat Protoc 1, 749–754 (2006). 17406304 10.1038/nprot.2006.101

[63] Salzmann M, Wider G, Pervushin K, Senn H, Wüthrich K, TROSY-type Triple-Resonance Experiments for Sequential NMR Assignments of Large Proteins. J Am Chem Soc 121, 844–848 (1999).

[64] Eletsky A, Kienhofer A, Pervushin K, TROSY NMR with partially deuterated proteins. J Biomol NMR 20, 177–180 (2001). 11495249 10.1023/a:1011265430149

[65] Shen Y, Delaglio F, Cornilescu G, Bax A, TALOS+: a hybrid method for predicting protein backbone torsion angles from NMR chemical shifts. J Biomol NMR 44, 213–223 (2009). 19548092 10.1007/s10858-009-9333-z PMC2726990

[66] Berjanskii MV, Wishart DS, A simple method to predict protein flexibility using secondary chemical shifts. J Am Chem Soc 127, 14970–14971 (2005). 16248604 10.1021/ja054842f

[67] Ulrich EL , BioMagResBank. Nucleic Acids Res 36, D402–408 (2008). 17984079 10.1093/nar/gkm957 PMC2238925

[68] Mulder FA, Schipper D, Bott R, Boelens R, Altered flexibility in the substrate-binding site of related native and engineered high-alkaline Bacillus subtilisins. J Mol Biol 292, 111–123 (1999). 10493861 10.1006/jmbi.1999.3034

[69] Virtanen P , SciPy 1.0: fundamental algorithms for scientific computing in Python. Nat Methods 17, 261–272 (2020). 32015543 10.1038/s41592-019-0686-2 PMC7056644

[70] Newville M , LMFIT: non-linear least-square minimization and curve-fitting for Python, http:/lmfit.github.io/lmfit-py. (Zenodo, 2020, doi: 10.5281/zenodo.598352).

[71] Foreman-Mackey D, Hogg DW, Lang D, Goodman J, emcee: The MCMC Hammer. Publ Astron Soc Pac 125, 306–312 (2013).

[72] Helmus JJ, Jaroniec CP, Nmrglue: an open source Python package for the analysis of multidimensional NMR data. J Biomol NMR 55, 355–367 (2013). 23456039 10.1007/s10858-013-9718-x PMC3636164

[73] Iranpoor N, Firouzabadi H, Nowrouzi N, A novel method for the highly efficient synthesis of 1,2-benzisoxazoles under neutral conditions using the Ph3P/DDQ system. Tetrahedron Lett 47, 8247–8250 (2006).

[74] Johnson KA, Fitting enzyme kinetic data with KinTek Global Kinetic Explorer. Methods Enzymol 467, 601–626 (2009). 19897109 10.1016/S0076-6879(09)67023-3

[75] Johnson KA, Simpson ZB, Blom T, Global kinetic explorer: a new computer program for dynamic simulation and fitting of kinetic data. Anal Biochem 387, 20–29 (2009). 19154726 10.1016/j.ab.2008.12.024

[76] Gontero B, Meunier JC, Buc J, Ricard J, The 'slow' pH-induced conformational transition of chloroplast fructose 1,6-bisphosphatase and the control of the Calvin cycle. Eur J Biochem 145, 485–488 (1984). 6096140 10.1111/j.1432-1033.1984.tb08582.x

[77] Johnson KA, Simpson ZB, Blom T, FitSpace explorer: an algorithm to evaluate multidimensional parameter space in fitting kinetic data. Anal Biochem 387, 30–41 (2009). 19168024 10.1016/j.ab.2008.12.025

