# Supplementary material for: How directed evolution reshapes energy landscapes in enzymes to boost catalysis
Source: Science. Author manuscript; Available in PMC 2022 Oct 28. (PMC9616100; doi:10.1126/science.abd3623)
Supplement: 1 [file NIHMS1842694-supplement-1.pdf]

Supplementary Materials for

How directed evolution reshapes energy landscapes in enzymes to boost catalysis

Renee Otten, Ricardo A. P. Pádua, H. Adrian Bunzel, Vy Nguyen, Warintra Pitsawong, MacKenzie Patterson, Shuo Sui, Sarah L. Perry, Aina E. Cohen, Donald Hilvert*, Dorothee Kern*

Correspondence to: hilvert@org.chem.ethz.ch or dkern@brandeis.edu

This PDF file includes:

Materials and Methods
Figs. S1 to S19
Tables S1 to S3
References (36-77)

Materials and Methods

Protein expression and purification

Initial experiments (i.e., Fig. 1B-D and NMR backbone assignment) were performed with the constructs described by Blomberg *et al.*, containing a C-terminal His_6-tag (*3*). While the His_6-tag can be cleaved with Factor Xa protease, it leaves an additional six residues (GSIEGR) at the C-terminus. To assert that these residues do not affect any of the observed behavior (and for consistency between all constructs), we switched to a Tobacco Etch Virus (TEV) protease-cleavable, N-terminal His-tag (see below), which only leaves three residues (GSG) in front of the native protein sequence. None of the measured biochemical properties (e.g., catalytic activity and thermal-shift assay based stability measurements) are affected by this change and only local perturbations near the N- and C-terminus are observed in the NMR spectra (Fig. S1B).

Genes for wild-type proteins were codon-optimized, synthesized, and sub-cloned by Genscript (Piscataway, NJ; DNA sequences in Table S2). An N-terminal His_6-tag, followed by a TEV-cleavage site, was added to the genes for HG3.7 and HG3.17 and the resulting construct was sub-cloned into pET-11b. The gene for HG3 was sub-cloned into pETM-41, which already contained a His_6-tag, with an added MBP-tag (for expression/solubility reasons) followed by a TEV cleavage site at the N-terminus. For ease of comparison, the same residue numbering as in the earlier HG3.17/TSA complex (PDB 4bs0) (*3*) is used throughout the manuscript.

Kemp mutants were generated using site-directed mutagenesis on the wild-type plasmids using the QuickChange Lightning kit (Agilent Technologies, Santa Clara, CA) with the primer sequences (obtained from Integrated DNA Technologies, Croalville, IA) listed in Table S3. The resulting DNA was sequenced to verify correctness using Sanger sequencing by Genewiz (Cambridge, MA, USA) with standard T7-, T7-term, and the sequencing primers listed in Table S3.

Escherichia coli BL21(DE3) competent cells (New England Biolabs, Ipswich, MA) were transformed and used for protein expression either in Terrific Broth (TB; Thermo Fisher Scientific, Waltham, MA) or M9 minimal media for isotopically-labelled proteins. M9 minimal media contained 1 g/L $^{15}NH_4Cl$ and 10 g/L D-glucose (for ^{15}N-labeled proteins) or was prepared in D_2O containing 1 g/L $^{15}NH_4Cl$ and 2 g/L U-[^{13}C,^{2}H]-D-glucose for $^{2}H/^{13}C/^{15}N$-labeled proteins. All isotopically labeled compounds were obtained from Cambridge Isotope Laboratories, (Tewksbury, MA). Briefly, a 20-50 mL starter culture from a single colony was grown overnight at 30 °C and used to inoculate 1 L of media in a 2.8 L Fernbach flask. Cells were initially grown at 37 °C and protein expression was induced by adding 250 µM (1 mM for wild-type HG3 and mutants) IPTG at mid log phase of growth (OD_{600} of 1.2 for TB and 0.8 for M9) and carried out overnight at 20 °C (18 °C for wild-type HG3 and its single- and double mutants). Cells were harvested by centrifugation at 4000 x g and stored at -80 °C until purification.

Cell pellets were resuspended in buffer A (50 mM Tris-HCl, pH 7.5, and 500 mM NaCl), containing 1x Halt Protease Inhibitor Cocktail (Thermo Fisher Scientific, Waltham, MA) and lysozyme and DNAse I (Sigma-Aldrich, St. Louis, MO). For HG3 and its mutant forms, 0.5 mM EDTA (Thermo Fisher Scientific, Waltham, MA) was used during lysis and 1 mM β-mercaptoethanol (from the Halt Protease Inhibitor Cocktail kit) and 10% (v/v) glycerol was present throughout the purification. After sonication for 10 minutes, using 30 W pulses of 20 s with 30 s resting intervals on ice, the lysate was centrifuged for 45 min at 30,000 x g at 4 °C. The soluble fraction was applied onto a HisTrap column (GE Healthcare, Chicago, IL) pre-equilibrated with

buffer A. Impurities were washed off with 5 column volumes of buffer A containing 30 mM imidazole (15 mM imidazole for wild-type HG3 and its mutants) and protein was eluted using buffer A with the addition of 300 mM imidazole. The N-terminal tags were cleaved by an in-house prepared His-tagged TEV protease (1:20 TEVP:Kemp) concomitantly with dialysis in buffer A overnight at 4 °C. A second HisTrap purification was carried out and tag-free Kemp eliminase was recovered in the flow-through. Size-exclusion chromatography was carried out in 50 mM sodium phosphate, pH 7.0, and 100 mM NaCl (with the addition of 2 mM TCEP and 10% (v/v) glycerol for HG3) using a HiLoad 16/600 Superdex 75 pg or HiLoad 16/600 Superdex 200 pg column (GE Healthcare, Chicago, IL). Fractions containing pure Kemp eliminase were pooled, concentrated, flash-frozen in liquid nitrogen, and stored at -80 °C until further use. All purification steps were performed at 4 °C.

Thermal-shift assay measurements to determine protein stability
Melting curve analyses were performed with 20 µL samples containing 10 µM protein in 50 mM sodium acetate, 50 mM bis-Tris propane, and 100 mM NaCl at varying pH with the thermal shift assay kit (Thermo Fisher Scientific, Waltham, MA). Melting curves between 20 °C and 99 °C were recorded with a StepOnePlus Real-Time PCR thermocycler (Thermo Fisher Scientific, Waltham, MA) according to the user's manual. Figures were prepared with Python v3.8 (Python Software Foundation, https://www.python.org) using the NumPy (*36*) and matplotlib (*37*) packages.

X-ray crystallography
Crystallization. Initial screenings were conducted at 18 °C, unless otherwise stated, using the sitting drop vapor diffusion method. Kemp eliminase variants at 10 mg/mL (in 5 mM sodium phosphate, pH 7, and 100 mM NaCl) were mixed with solutions (0.5 µL:0.5 µL) of commercially available crystallization kits (Hampton Research, Aliso Viejo, CA; Jena Bioscience, Jena, Germany; Qiagen, Germantown, MD) by a Crystal Phoenix liquid handler (Art Robbins, Sunnyvale, CA) in a 96-well INTELLI-PLATE® (Art Robbins, Sunnyvale, CA). Co-crystallization with TSA was achieved by adding 100 mM TSA dissolved in DMSO to the protein stock (final concentrations: 2 mM for HG3 and HG3 K50Q; 5 mM for HG3.7; and 2.5 mM for HG3.17). Paratone-N (Hampton Research, Aliso Viejo, CA) was the cryoprotectant of choice with the exceptions indicated below.

- HG3 was crystallized at 4 °C in 0.2 M magnesium chloride hexahydrate, 0.1 M HEPES, pH 7.5, 25% (w/v) polyethylene glycol (PEG) 3,350. The crystal was bathed in a cryoprotectant solution (3 µL of reservoir solution and 1µL of glycerol) and flash cooled in liquid nitrogen.
- HG3 and HG3 K50Q with 2 mM TSA were crystallized in 2 M ammonium sulfate and 5% isopropanol.
- HG3 K50Q was crystallized in 2 M ammonium sulfate and 5% isopropanol.
- HG3.7 was crystallized in 100 mM Bis-Tris, pH 5.5, and 2 M ammonium sulfate.
- HG3.7 with 5 mM TSA was crystallized in 100 mM sodium acetate trihydrate, pH 4.6, and 2 M ammonium sulfate.
- HG3.17 was crystallized in 25% (w/v) PEG 4,000, 100 mM MES, pH 6.5, and 200 mM magnesium chloride.

- HG3.17 E47N/N300D was crystallized in 30% (w/v) PEG 6,000, 100 mM PIPES, pH 7.0, and 10 mM DTT. LV CryoOil (MiTeGen, Ithaca, NY) was used as cryoprotectant. Except for the mutation sites, the HG3.17 and HG3.17 E47N N300D structures are identical.
- HG3.17 E47N/N300D with 2.5 mM TSA was crystallized in 100 mM sodium acetate, pH 4.6, and 2 M ammonium sulfate.

To obtain the inactive structure of HG3.17, crystallization screens were performed using the protein dialyzed in 380 mM Bis-Tris propane, pH 10 at 37 °C. Out of 400 conditions tested only 11 yielded protein crystals. Calcium was the only common element in these formulations. Thus, a second round of screening was carried out with 6 mg/mL HG3.17 dialyzed in 380 mM Bis-Tris propane, pH 10, and 200 mM calcium chloride. Crystals were obtained in 2.1 M malic acid, pH 7.

Of note, the published structure of the TSA-bound form of HG3.17 E47N/N300D (PDB 4bs0) presented a rigid-body displacement in one of the two molecules in the asymmetric unit (*3*). In our new crystallization conditions, there is only one molecule in the asymmetric unit without the rigid-body displacement. In addition, the structures of wild-type HG3.17 and HG3.17 E47N/N300D are virtually identical. However, crystals for the mutant are bigger, easier to grow and diffract at higher resolution. Therefore, the crystal structures of HG3.17 presented here were all derived from the HG3.17 E47N/N300D with the exception of the calcium-bound HG3.17 structure.

Data collection and processing. Cryogenic (100 K) single-crystal X-ray diffraction data were collected at beamlines 8.2.1 and 5.0.1 at Advanced Light Source (ALS) and beamline 14-1 at the Stanford Synchrotron Radiation Lightsource (SSRL). To better understand the structural nature of the inactive state, diffraction data were collected at 343 K at the SSRL beamline 7-1. The temperature was regulated by a Cryostream 800 controller (OxfordCryosystems, Oxford, United Kingdom), and a home-built sample holder was used to prevent crystal dehydration during the experiment. The data were integrated in iMosflm (*38*) or XDS (*39*), scaled and merged in Aimless (*40*), and the data quality assessed with the Xtriage software available in Phenix (*41*). Structures were solved by molecular replacement using the chain A of PDB 4bs0 (*3*) as a search model in Phaser (*42*).

Refinement and model building. Single-model structures were refined in phenix.refine (*43*) interspersed with manual building cycles using *Coot* (*44*). To model the conformational heterogeneity observed in the HG3.17 data set collected at 343 K, the active conformation, corresponding to the structure of the TSA-bound HG3.17, and the inactive structure, corresponding to the calcium bound HG3.17, were joined in a single chain as different alternate conformations. The refinement was carried out by grouping the occupancies for each alternate conformation.

Ensemble refinement. The conformation flexibility of Kemp-TSA complexes was probed by fitting structure ensembles generated by molecular dynamics to the X-ray data as implemented in phenix.ensemble_refinement (*18*). Harmonic restraints for all ligands were used during refinement. Default values for wray_coupled_tbath_offset (5.0) and pTLS values (0.6, 0.8, 0.9, 1.0) were tested and the relaxation time Tx automatically selected according to the resolution. The solution with the lowest R_{free} was chosen as recommend by the developers.

The model placement in the unit cell was standardized using ACHESYM (*45*). Macromolecular representations were rendered in PyMOL (*46*) and secondary structure elements were calculated using DSSP (*47, 48*). Maximum likelihood structural superpositions were performed in THESEUS (*49*) and interatomic interactions in protein structures were calculated using Arpeggio (*50*). The real-space difference density *Z* score (RSZD) was calculated using EDSTATS implemented in CCP4 (*51*) and the calcium binding site coordination was validated using the CheckMyMetal server (*52*).

NMR spectroscopy and data analysis

NMR experiments were recorded on an Agilent DD2 600 MHz or a Bruker Avance II 800 MHz four-channel spectrometer, both equipped with a triple-resonance cryogenically cooled probe-head. Unless otherwise stated, NMR samples contained 0.2–1.0 mM Kemp eliminase in 50 mM sodium phosphate, pH 7.0, 100 mM NaCl and 10% (v/v) D_2O. Sample temperatures were calibrated using the "4% methanol + 96% methanol-*d*4" and "80% ethylene glycol in DMSO-*d*6" samples (Cambridge Isotope Laboratories, Tewksbury, MA).

All data sets were processed with the NMRPipe/NMRDraw software package (*53*) and visualized/analyzed using the programs NMRFAM-SPARKY (*54*) and/or PINT (*55*). Briefly regarding the processing, an appropriate linear prediction algorithm (i.e., mirror image linear prediction (*56*) for constant-time evolution or forward-backward linear prediction (*57*) in the case of real-time evolution) was employed to double the indirect domains. A cosine-squared window function was employed in all domains. All chemical shifts are referenced to DSS according to Wishart *et al.* (*53*). Figures were prepared with Python v3.8 (Python Software Foundation, https://www.python.org) using the NumPy (*36*) and matplotlib (*37*) packages.

Backbone assignments for HG3.17. Backbone assignment experiments were recorded at 27 °C on a 1 mM $^{2}H/^{13}C/^{15}N$-labeled HG3.17 sample in 100 mM HEPES, pH 7.0, 50 mM NaCl. TROSY-versions of a 3D HNCA, HN(CO)CA, HNCACB, HN(CO)CACB, HN(CA)CO, and HNCO experiments (*58*), a 3D ^{15}N-edited NOESY-HSQC (non-TROSY) (*59*), and a 2D [^{1}H, ^{15}N]-TROSY-HSQC (*60, 61*) experiment were recorded on an Agilent DD2 600 MHz spectrometer.

During the initial round of the backbone assignment we noticed that most of our assigned residues were located in loops and α-helices, with hardly any coverage close to and inside the β-barrel interior of Kemp eliminase. We conjectured that this could be caused by incomplete back-exchange of amide positions from deuterium (since D_2O was used in the expression media) to protons during the purification process, likely due to hydrogen-bonding interactions. We, therefore, added a mild unfolding-refolding step using guanidine hydrochloride following Tugarinov *et al.* (*62*) to our purification protocol (only for the deuterated sample), which did not affect enzymatic activity. In short, HG3.17 in 100 mM HEPES, pH 7.0, and 50 mM NaCl was mixed with denaturing buffer (20 mM Tris, pH 7.0, 100 mM NaCl, 6 M guanidine hydrochloride) to give a final protein concentration of 1.5 mg/mL, and was subsequently incubated for 1 hour at room temperature. To start the refolding step, an appropriate volume of refolding buffer (20 mM Tris, pH 7.0, 5 mM $MgSO_4$, 2 mM benzamidine, 0.4 M L-Arg) was cooled to 4 °C and under stirring the protein solution was dropwise added so that the final protein concentration was 35 μg/mL. The sample was incubated for 2 hours at room-temperature before performing the final size-exclusion chromatography step. Indeed, we observed higher intensities for several cross peaks in the 2D NMR experiments and the appearance of cross peaks indicative of a more complete back-exchange.

Subsequently, we repeated some of the NMR assignment experiments on this back-exchanged sample: 3D HNCACB and HN(CO)CACB experiments (*63, 64*) on the Bruker Avance II 800 MHz spectrometer, and 3D HNCA, HNCO, and HN(CA)CO experiments (*58*) on the Agilent DD2 600 MHz spectrometer. We indeed observed more (intense) cross peaks in our 3D NMR experiments and could assign more cross peaks residue-specifically, nevertheless much the β-barrel interior remains unassigned. The assigned chemical shifts were corrected for deuterium isotope effects and used as input for the program TALOS+ (*65*), which includes the calculation of order parameters from chemical shifts using the 'RCI' method by Berjanskii and Wishart (*66*). The chemical shift assignment for HG3.17 has been deposited in the BioMagResBank (*67*) with accession code 50471.

Temperature- and pH-dependence of the active/inactive equilibrium in free HG3.17. NMR samples contained ~1 mM ^{15}N-labeled HG3.17 and 10% (v/v) D_2O with TMSP as internal standard for referencing. At pH 4, the buffer consisted of 100 mM sodium acetate, and 50 mM NaCl whereas 100 mM MOPS and 50 mM NaCl was used at either pH 7.0 or 9.0. Two-dimensional [^{1}H, ^{15}N]-TROSY-HSQC (*60, 61*) spectra were recorded for 1.25 h at temperatures between 5 and 40 °C in 5 °C intervals after equilibrating the sample temperature for 30 minutes.

NMR titrations. To verify the three-step binding model suggested by the stopped-flow experiments for HG3.17 and the TSA, an NMR titration experiment was performed with 500 µM ^{15}N-labeled HG3.17 and TSA (0 – 1 mM concentration) at 27 °C and 40 °C. Dissociation constants for HG3 and TSA or HG3.17 and product were obtained from [^{1}H, ^{15}N]-TROSY-HSQC (*60, 61*) spectra with ^{15}N-labeled variants (200 µM for HG3 and 500 µM for HG3.17) and increasing amounts of TSA (0 – 1 mM) or product (0 – 50 mM) at 25 °C. Line-shape fitting was performed using PINT (*55*) to obtain cross-peak positions in the individual spectra. The combined chemical shift difference $\Delta\delta$ was calculated according to Equation 1:

$$\Delta\delta\ (ppm) = \sqrt{\left[\Delta\delta_{\mathrm{H}}{}^{2} + (\Delta\delta_{\mathrm{N}}/R_{\mathrm{scale}})^{2}\right]} \qquad \text{Equation 1}$$

where $\Delta\delta_{\mathrm{H}}$ and $\Delta\delta_{\mathrm{N}}$ are the chemical shift perturbations of the amide proton and nitrogen, respectively, and $R_{\mathrm{scale}} = 6.3$ was determined according to Mulder *et al.* (*68*). Resonances with a sufficient signal-to-noise ratio and for which $\Delta\delta \geq 0.015$ ppm were included to determine the dissociation constants using Equation 2:

$$\Delta\delta = \Delta\delta_{\mathrm{sat-free}} \cdot \frac{[I]+[E_{\mathrm{t}}]+K_{\mathrm{D}}-\sqrt{([I]+[E_{\mathrm{t}}]+K_{\mathrm{D}})^{2}-4\cdot[E_{\mathrm{t}}]\cdot[I]}}{2\cdot[E_{\mathrm{t}}]} \qquad \text{Equation 2}$$

where $\Delta\delta_{\mathrm{sat\text{-}free}}$ is the combined chemical shift difference between free and saturated protein, $[I]$ is the concentration of TSA or product, and $[E_{\mathrm{t}}]$ is the total enzyme concentration. All resonances 21 for HG3 + TSA and 15 for HG3.17 with product, respectively) were fit simultaneously and plotted using Python v3.8 (Python Software Foundation, https://www.python.org) using the NumPy (*36*), SciPy (*69*), matplotlib (*37*), lmfit (*70*), and emcee (*71*) packages. Error bars denote the standard errors as obtained from the global fit using the emcee algorithm. The populations of the two TSA-bound species were calculated from the peak volumes as described below, and 17 (20) residues were included at 25 °C (40 °C).

Effect of calcium on active/inactive equilibrium. To verify the effect of Ca^{2+} on the active/inactive populations in solution, we recorded 2D [^{1}H, ^{15}N]-TROSY-HSQC (*60, 61*) spectra of free and calcium-bound ^{15}N-labeled HG3.17. The protein was dialyzed overnight at 4 °C into 50 mM bis-Tris propane, pH 7.0, 100 mM NaCl, and samples contained 600 µM protein and 10% (v/v) D_2O. Samples were equilibrated for at least 5-8 hours at the measurement temperature before recording the NMR data. After recording the NMR spectra at 25 °C in the absence of calcium, the sample was buffer exchanged into 50 mM bis-Tris propane, pH 7.0, 100 mM NaCl, containing 100 mM $CaCl_2$ and the experiment was repeated. One-dimensional projections onto the ^{15}N axis were generated using NMRPipe (*53*): the relevant part in the 2D spectrum was extracted and the data was projected onto an axis using the "proj.tcl" helper script.

Determination of populations. To determine the active/inactive populations in the free protein, 2D [^{1}H, ^{15}N]-TROSY-HSQC (*60, 61*) spectra were recorded (64 transients, interscan delay of 1 s, ~5h) for the Kemp eliminase variants. Samples contained 600 µM or 1 mM ^{15}N-labeled protein (for HG3/HG3.7 and HG3.17, respectively) in 50 mM sodium phosphate, 100 mM NaCl, 1% (v/v) DMSO, and 10% (v/v) D_2O. All proteins, in 5 mm Shigemi tubes, were equilibrated overnight at 25 °C before putting the sample in the magnet, which was already pre-equilibrated at the desired temperature, and starting the data acquisition. Afterwards, the NMR samples for HG3.7 and HG3.17 were incubated overnight at 40 °C before recording another 2D spectrum at this temperature; this was not possible for HG3 due to stability reasons. To determine the populations for the induced-fit step in HG3.17 with the TSA, experiments at 25 °C and 40 °C were performed as described above on a sample containing 500 µM of HG3.17 and 1 mM TSA. Line-shape fitting was performed using PINT (*55*) to obtain cross-peak volumes in the individual spectra. The population of the active/inactive species were calculated for residues with a sufficient signal-to-noise ratio and that are well enough separated to fit reliably. The minor, inactive, population for each variant was calculated from fitting all values (10, 7, and 3 residues for HG3, HG3.7, and HG3.17 at 25 °C, respectively; 15 and 23 residues for HG3.7 and HG3.17 at 40 °C, respectively) to a normal distribution and by calculating the weighted mean/standard deviation; both methods gave the same results within experimental error.

Role of His209 in the pre-existing equilibrium. The sample contained 1 mM ^{15}N-labeled HG3.17 H209A in 50 mM sodium phosphate, pH 7.0, 100 mM NaCl, and 10% (v/v) D_2O, and 2D [^{1}H, ^{15}N]-TROSY-HSQC (*60, 61*) spectra were recorded at 25 °C and 40 °C after several hours of equilibration at the desired temperature.

pH-jump experiments. ^{15}N-labeled protein (for 2D experiment on HG3.7) or unlabeled protein (for 1D experiment) was dialyzed overnight at 4 °C into 5 mM sodium phosphate, pH 10.0, and 100 mM NaCl. The sample was concentrated to ~1.5 mM in a total volume of 300 µL containing 20% (v/v) D_2O, and was transferred into a 5 mm Thin Wall Precision NMR Sample Tube 7"L (Wilmad-LabGlass, Vineland, NJ; product # 535-PP-7). All components were incubated at 25 °C for at least 5 hours before the start of the experiment, and tuning/matching, shimming, and pulse calibrations were performed on an already equilibrated sample with the identical, final buffer composition (i.e., after the mix with pH 7 buffer). Immediately before starting the NMR experiment, 300 µL buffer (50 mM sodium phosphate, pH 7.0, and 100 mM NaCl) was added to the NMR tube and gently mixed before inserting the sample in the magnet. A series of 2D [^{1}H, ^{15}N]-TROSY-HSQC (*60, 61*)

spectra with minimal phase cycle (~18 min per experiment) were recorded at 25 °C for HG3.7. One-dimensional proton experiments employing pre-saturation of the water signal were recorded with 16 transients, giving rise to a measurement time of 46 s for each experiment. Spectra were recorded continuously (256 experiments for HG3, 196 min; 128 experiments for HG3.7 and HG3.17, 98 min). The arrayed data was separated into individual 1D experiments with Python v3.8 (Python Software Foundation, https://www.python.org) using the nmrglue (*72*) package and processed with NMRPipe (*53*). Peak intensities were extracted and fitted simultaneously (5 peaks for HG3 and 3 peaks for HG3.7/HG3.17, respectively) to a single-exponential function, and plotted with Python v3.8 (Python Software Foundation, https://www.python.org) using the NumPy (*36*), SciPy (*69*), matplotlib (*37*), and lmfit (*70*) packages.

Substrate synthesis
5-Nitrobenzisoxazole was synthesized from the respective salicylaldehyde by first forming its oxime and subsequent cyclization with Ph_3P and DDQ according to published procedures (*73*).

Enzyme activity
Initial rate analysis. The k_{cat}/K_M values shown in Fig. 1C were determined using initial rate analysis for product formation. The temperature-dependent activity of the HG3 variants were analyzed on an SX.18MV stopped-flow instrument (Applied Photophysics, Leatherhead, Surrey, UK) by mixing protein (between 5 nM – 30 µM, final concentration) in 95 mM sodium phosphate, pH 7.5, and 200 mM NaCl in a 1:1 ratio with 5-nitrobenzisoxazole (50 µM, final concentration) in 5 mM sodium phosphate, pH 1.26, and 20% (v/v) methanol. After mixing the final substrate concentration was 25 µM in 50 mM sodium phosphate, pH 7.0, and 100 mM NaCl. The change in absorbance at 380 nm was measured for 1 s and converted to product formation using an extinction coefficient of 15,800 M^{-1} cm^{-1}.

Progress curves. Kemp eliminase variants were dialyzed in activity buffer (55.5 mM sodium phosphate, pH 6.82, and 111.1 mM NaCl) and incubated for 6 hours at 25 °C and degassed using ThermoVac (MicroCal, Northampton, MA) at 25 °C. 10x stocks of 5-nitrobenzisoxazole were prepared in methanol. 540 µL of enzyme was added to a quartz cuvette (Hellma, Plainview, NY) and a blank measurement was taken at 440 nm in a Cary 100 UV-Vis spectrophotometer (Agilent, Santa Clara, CA). 60 µL of 10x substrate was added to the cuvette and mixed, yielding a final concentration of enzyme (HG3: 16.6 µM; HG3 K50Q: 8.4 and 9.7 µM; HG3 M84C: 16.9 µM; HG3 K50Q/M84C: 600 nM; HG3.7: 300 nM; HG3.17: 66.67 nM) in activity buffer at pH 7.0.

Progress curves were measured until completion by following the product absorbance at 440 nm at 25 °C. The background reaction was measured in the absence of enzyme. The absolute substrate concentrations were obtained after diluting the completed reaction in a sodium hydroxide solution, measuring the absorbance at 380 nm and using the 15,800 M^{-1} cm^{-1} extinction coefficient (30 µL of the completed reaction was added to 540 µL of water and 30 µL of 0.6 M sodium hydroxide). A wavelength scan between 380 nm and 440 nm yielded the extinction coefficient to be used at 440 nm: 1,052.33 M^{-1} cm^{-1} (pH 7.0).

The data were analyzed in KinTek Explorer v9.0.200113 (KinTek Corporation, Snow Shoe, PA) (*74, 75*) to extract the steady-state parameters (see Fig. S14A for the scheme). In short, the rate for the background reaction (k_1) was fixed to its known value (k_1 = 1.04 x 10^{-4}) as well as the values

for k_2 and k_{-2}, which were determined by NMR (Fig. 2), and k_{-4} was set to zero as the reaction is irreversible. Of note, for the data fitting for HG3 K50Q, M84C, and K50Q/M84C we assumed that values for k_2 and k_{-2} are the same as for wild-type HG3. The on-rates for substrate and product (i.e., k_3 and k_{-5}, respectively) were set to diffusion-limited values of 1000 µM^{-1} s^{-1}, which left three fitting parameters (i.e., k_{-3}, k_4, and k_5). It is important to note that the individual microscopic rate constants cannot be determined, but the values for k_{cat}, K_M for substrate, K_S for substrate, and K_D for product can be estimated reliably using the equations below:

$$k_{\text{cat}} = \frac{k_4 k_5}{k_4 + k_{-4} + k_5} = \frac{k_4 k_5}{k_4 + k_5} \qquad \text{Equation 3}$$

$$K_{\text{M,substrate}} = \frac{k_4 k_5 + k_{-3} * (k_{-4} + k_5)}{\left(\frac{k_2 k_3}{k_{-2} + k_2}\right)(k_4 + k_{-4} + k_5)} = \frac{k_4 k_5 + k_{-3} k_5}{\left(\frac{k_2 k_3}{k_{-2} + k_2}\right)(k_4 + k_5)} \qquad \text{Equation 4}$$

$$K_{S,substrate} = \frac{k_{-3}}{k_3} \qquad \text{Equation 5}$$

$$K_{D,product} = \frac{k_{-5}}{k_5} \qquad \text{Equation 6}$$

Figures were prepared with Python v3.8 (Python Software Foundation, https://www.python.org) using the NumPy (*36*) and matplotlib (*37*) packages.

pH-rate profiles. The dependence of k_{cat}/K_M on pH was determined for HG3.17 at 20 °C and enzymes were preincubated for at least 30 min in buffer at the final pH. Acetate buffer (50 mM sodium acetate and 100 mM NaCl) was employed for pH values from pH 4 to 5.5 and bis-Tris propane buffer (50 mM bis-Tris propane and 100 mM NaCl) from pH 6 to 9.5. The background rate without enzyme was determined for each pH value and subtracted. The extinction coefficient was corrected according to the following equation using a product pK_a of 3.98 and $\Delta\varepsilon_{max}$ of 15,800 M^{-1} cm^{-1}.

$$\Delta\varepsilon = \frac{\Delta\varepsilon_{\text{max}}}{1 + 10^{\text{p}K_{\text{a}} - \text{pH}}} \qquad \text{Equation 7}$$

pH-jump experiments followed by activity measurements and Trp fluorescence.
Activity-based pH-jump experiments were performed on a Lambda 35 UV/Vis spectrometer (PerkinElmer, Schwerzenbach, Switzerland). After preincubation of 10 µM HG3 or 1 µM HG3.17 at 20 °C in buffer (50 mM bis-Tris propane, 50 mM sodium acetate, and 100 mM NaCl) at pH 4.0 or 10.0, the enzymes were diluted 1:100 into the assay buffer (250 mM sodium acetate, pH 5.0, and 100 mM NaCl) supplemented with 10% (v/v) methanol and a final concentration of 5-nitrobenzisoxazole of 100 µM. Under these conditions, substrate conversion was below 10% after 1 h incubation with HG3 or 15 min incubation with HG3.17.

Tryptophan fluorescence-based pH-jump experiments were performed with a spectrofluorometer (Photon Technology International, Birmingham, NJ). Ten micromolar of HG3 or HG3.17 were preincubated at 20 °C in buffer (50 mM bis-Tris propane, 50 mM sodium acetate, and 100 mM NaCl) at pH 4.0 or 10.0. Upon excitation at 280 nm, the 310 nm to 360 nm emission ratio was recorded. Changes in fluorescence were recorded upon 1:10 dilution of the protein in buffer (50 mM bis-Tris propane, 50 mM sodium acetate, and 100 mM NaCl) at varying pH values.

Fluorescence intensity was recorded alternatingly for 1 s at each wavelength and afterwards the shutter was closed for 1 s to prevent photobleaching. The final pH after dilution was measured for the analysis of the pH dependence and the data were fitted to the following equation (*76*):

$$k_{\text{obs}} = \frac{k_1 K_{\text{act}} + k_2[\text{H}]}{K_{\text{act}} + [\text{H}]} + \frac{k_{-2}\frac{K_{\text{act}} k_1}{k_2} + k_{-2}k_{-1}[\text{H}]}{K_{\text{inact}} + [\text{H}]} \quad \text{Equation 8}$$

K_D measurements for the transition-state analogue

Overall dissociation constants for the transition-state analogue (TSA; 6-nitrobenzotriazole) to HG3.7 and HG3.17 were measured using Trp fluorescence titration experiments at different temperatures. The TSA was purchased from Millipore Sigma. One stock solution of 100 mM 6-nitrobenzotriazole in 100% DMSO was prepared, aliquoted, and stored at -20 °C until further use. Dilutions were made in several steps to slowly decrease the DMSO concentration, with buffer without DMSO (50 mM phosphate, pH 7.0, and 100 mM NaCl) to a final concentration of 1 mM TSA in 1% (v/v) DMSO. From here, serial dilutions were prepared in the same buffer containing 1% (v/v) DMSO. Experiments below room-temperature were performed in a 50 µL cuvette using the FluoroMax-4 spectrofluorometer (Horiba Scientific, Piscataway, NJ), whereas experiments above were done in a 96-well plate (Corning, Corning, NY; product #3994) using the SpectraMax® i3x *Multi-Mode* Microplate Reader (Molecular Devices, San Jose, CA). As a control, experiments at 20 °C were carried out using both methods to verify that the two methods give the same results within experimental error.

Samples were prepared individually to minimize photo-bleaching and contained 1 µM HG3.7 or HG3.17 and increasing amounts of the TSA (0 – 150 µM) in 50 mM phosphate, pH 7.0, 100 mM NaCl, and 1% (v/v) DMSO. At lower temperature (5, 10, and 15 °C), 200 nM of HG3.17 was used with TSA concentrations varying between 0 and 20 µM in the same buffer. After incubating the samples overnight at the required temperature, Trp fluorescence was measured using an excitation wavelength of 295 nm (9 and 5 nm bandwidth for the plate-reader and spectrofluorometer, respectively) and emission spectra were recorded from 320 to 600 nm (15 and 5 nm bandwidth for the plate-reader and spectrofluorometer, respectively) in increments of 2 nm. Control experiments with increasing TSA in buffer indicate that significant inner-filter effects (both at the excitation and emission wavelengths) are present at high TSA concentrations. For this reason, the affinity of the TSA to HG3 cannot be reliably determined from Trp fluorescence, and NMR titrations experiments were employed instead (see above).

The fluorescence intensity at 350 nm versus the TSA concentration was fitted to Equation 9 to obtain the K_D and plotted with Python v3.8 (Python Software Foundation, https://www.python.org) using the NumPy (*36*), SciPy (*69*), matplotlib (*37*), lmfit (*70*), and emcee (*71*) packages. Error bars denote the standard errors as obtained from the fit using the emcee algorithm.

$$F = F_0 + A \cdot \frac{[I] + [E_t] + K_D - \sqrt{([I] + [E_t] + K_D)^2 - 4 \cdot [E_t] \cdot [I]}}{2 \cdot [E_t]} \quad \text{Equation 9}$$

where F and F_0 are the fluorescence and initial fluorescence intensities, respectively; $[I]$ and $[E_t]$ are the concentration of TSA and total enzyme concentration, respectively.

Stopped-flow experiments with the transition-state analogue

Stopped-flow experiments measuring intrinsic tryptophan fluorescence were used to monitor the kinetics of TSA binding to the Kemp eliminases. The measurements were performed using an SX-20 stopped-flow instrument (Applied Photophysics, Leatherhead, Surrey, UK), equipped with a water bath to control the temperature. Changes in the intrinsic Trp fluorescence upon binding and dissociation of the TSA were monitored using an excitation wavelength of 295 nm (2.3 nm bandwidth) and a long-pass 320 nm cut-off filter to detect emission. All experiments were performed at 25 °C, except for HG3 where the kinetics were too fast and the temperature was lowered to 10 °C instead. One stock solution of 100 mM 6-nitrobenzotriazole in 100% DMSO was prepared, aliquoted, and stored at -20 °C until further use. Dilutions were made in several steps to slowly decrease the DMSO concentration, with buffer without DMSO (50 mM phosphate, pH 7.0, 100 mM NaCl) until a final concentration of 1 mM TSA in 1% (v/v) DMSO was reached. From here, serial dilutions were prepared in the same buffer containing 1% (v/v) DMSO.

In short, degassed buffer (50 mM sodium phosphate, pH 7.0, 100 mM NaCl, and 1% DMSO) was used to rinse the flow system and make it anaerobic in order to minimize photobleaching. The stock solutions of Kemp eliminases and TSA were made anaerobic by degassing using ThermoVac (MicroCal, Northampton, MA) at the desired temperature. For binding experiments, a solution of 5 μM HG3, HG3.7, or HG3.17 was loaded in the small syringe and quickly mixed with varying concentrations of TSA, prepared in the same buffer, loaded in the large syringe (mixing ratio 1:10; resulting in a final enzyme concentration in the observation cell of 0.45 μM). For dilution experiments, the pre-equilibrated, equimolar Kemp:TSA complex (50 μM for HG3 and 5 μM for HG3.7 and HG3.17) was loaded in the small syringe and diluted 11-fold with buffer in the large syringe. A significant decrease or increase in the fluorescence intensity of Kemp eliminase variants can be observed due to the binding or release of the TSA, respectively. At least five replicate measurements were made for each TSA concentration and the resulting traces were averaged using the Pro-Data Viewer software (Applied Photophysics, Leatherhead, Surrey, UK). We determined experimentally that no photo-bleaching correction was needed at the short measurement times used here (<1 s).

The individual traces were fitted to exponential equations and plotted using an in-house script for Python v3.8 (Python Software Foundation, https://www.python.org) that utilizes the NumPy (*36*), SciPy (*69*), matplotlib (*37*), lmfit (*70*), and emcee (*71*) packages. Error bars denote the standard errors as obtained from the fit using the emcee algorithm. All kinetic data were globally fitted in KinTek Explorer v9.0.200113 (KinTek Corporation, Snow Shoe, PA) (*74, 75*), after correcting for the dead-time of the instrument (2.5 ms). The interconversion between binding-competent and binding-incompetent forms of Kemp eliminase variants is orders of magnitude slower than the time traces measured here and, therefore, effectively no interconversion takes place during our stopped-flow experiments. For HG3.7 and HG3.17, we used the populations and interconversion rates determined from our NMR data at 25 °C to establish the fractions of active/inactive at the start of the stopped-flow experiment. The NMR data for HG3 recorded at 5 °C shows no indications of an "inactive" species, as expected at low temperatures, and we conjecture that all HG3 molecules are in the binding-competent state at 10 °C as well. To assess how well the parameters are constrained by the data and get more accurate uncertainties on the fitted parameters, we used the FitSpace Explorer feature (*77*) as implemented in KinTek Explorer (*74, 75*). As described in the KinTek Explorer manual and a recently published book (*17*), one can -at least in theory- calculate a χ^2 threshold that would represent the 95% confidence interval. However, as stated by the authors this is complicated in practice by the invalid assumption that

data points in the time traces are "independent and identically distributed points". Therefore, we follow their recommendation to use a 5-10% increase in the χ^2 to find lower and upper limits for a parameter that represent the 95% confidence interval, which represents 2σ. In this work, we set the χ^2 threshold to 0.95 and calculated the 1σ-uncertainties in the fitted parameters as:

$$\sigma = \frac{(\text{upper}_{\text{limit}} - \text{lower}_{\text{limit}})}{2 \cdot 1.96} \quad \text{Equation 10}$$

Overall dissociation constants are calculated from the microscopic rate constants according to Equation 11 (conformational selection + binding, for HG3.7) or Equation 12 (conformational selection + binding + induced-fit step, for HG3.17):

$$K_\text{D} = (K_1 + 1) * K_2 \quad \text{Equation 11}$$

$$K_\text{D} = \frac{(K_1 + 1) * K_2 * K_3}{K_3 + 1} \quad \text{Equation 12}$$

where K_1 (CS) = k_{-1} / k_1; K_2 (binding) = k_{-2} / k_2 = k_{off} / k_{on}, and K_3 (IF) = k_{-3} / k_3.

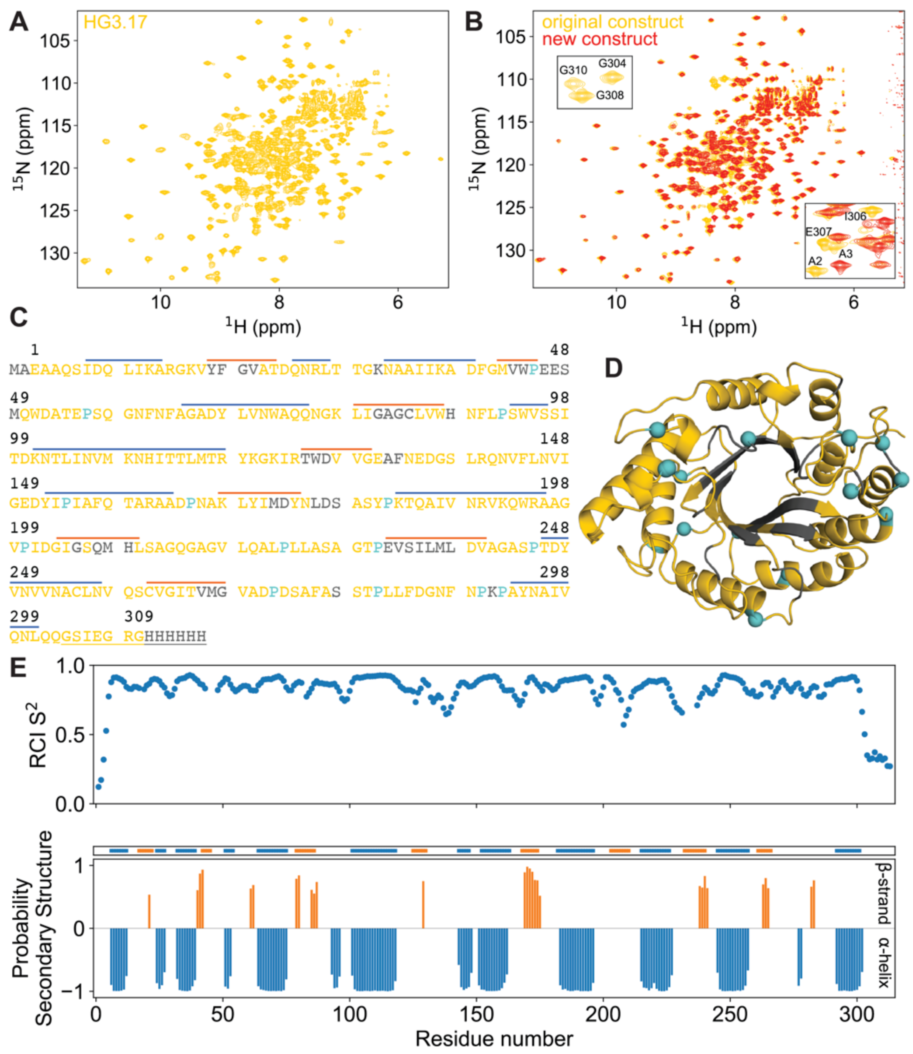

Fig. S1. Backbone assignment of HG3.17 and chemical shift analysis. (**A, B**) 2D [^{1}H, ^{15}N]-TROSY-HSQC spectrum of HG3.17 (original construct) recorded in 100 mM HEPES, pH 7.0, 50 mM NaCl at 27 ºC (**A**). The construct from Blomberg *et al.* (*3*) that contains a C-terminal His$_6$-tag was used to obtain backbone assignments and the data presented in Fig. 1B. To make sure that the His$_6$-tag does not affect our results, all later experiments were performed on a construct that does not contain the C-terminal His$_6$-tag, and after cleavage of a newly introduced N-terminal His$_6$-tag in our new construct (see Methods). (**B**) The NMR spectra for the original and His$_6$-tag cleaved constructs are virtually identical, as are their respective activities. Cross peaks with significant changes are shown in the insets, and correspond to residues in the N- and C-terminal region where the two constructs indeed differ. (**C, D**) Results obtained from the backbone assignment for the original construct are shown on the amino acid sequence with a color bar indicating the secondary structure element (blue/orange for helix and strand, respectively) (**C**) and plotted on the structure of free HG3.17 (**D**). Assigned residues are color-coded in yellow, unassigned and Pro residues are shown in gray and cyan, respectively. (**E**) The predicted order parameters from chemical shifts reveal that only the termini are flexible (upper panel). The NMR prediction of secondary structure elements agrees well with the crystallographic data as indicated by the color bar above the probability (lower panel).

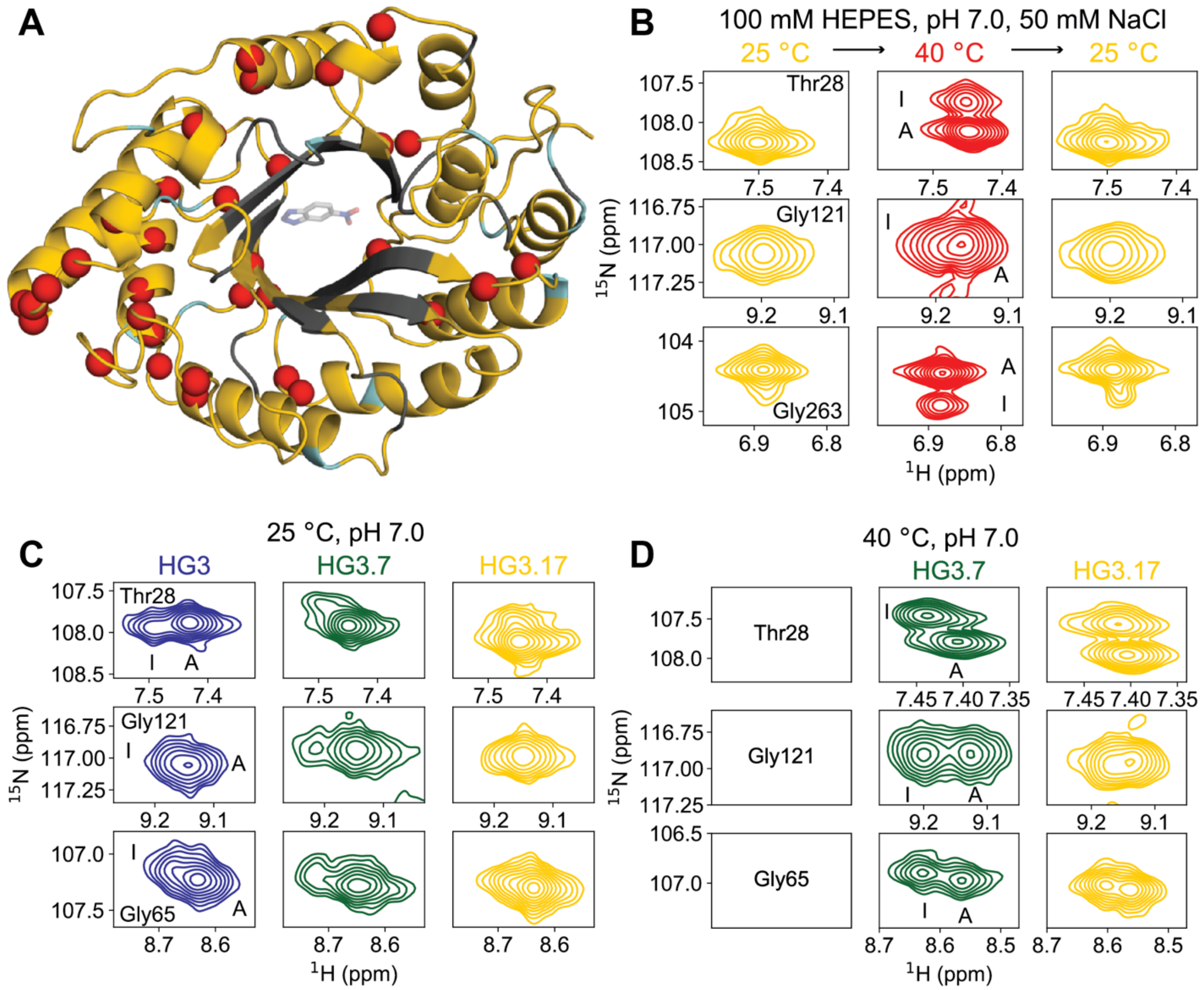

Fig. S2. Peak duplication is observed for all HG3 variants and the inactivation process is reversible. (**A**) Residues that show peak duplication in the NMR spectra of HG3.17 as a result of the active-inactive equilibrium in HG3.17 are shown as red spheres on the crystal structure of free HG3.17. Unassigned and Pro residues are indicated in gray and cyan, respectively. (**B**) The inactive/active equilibrium is strongly temperature dependent, with a larger fraction in the inactive conformation at high temperature. The inactivation process is, however, completely reversible as shown by the identical spectra recorded at 25 ºC before (left) and after (right) recording data at 40 ºC (middle). (**C, D**) Similar to HG3.17, peak duplication is present in HG3 and HG3.7 at 25 ºC (**C**) and becomes more pronounced at elevated temperatures for HG3.7 (40 ºC, **D**). The data were recorded in 50 mM sodium phosphate, pH 7.0, 50 mM NaCl, and 1% (v/v) DMSO. Of note, it was not possible to record NMR data for HG3 at 40 ºC due to its lower T_m.

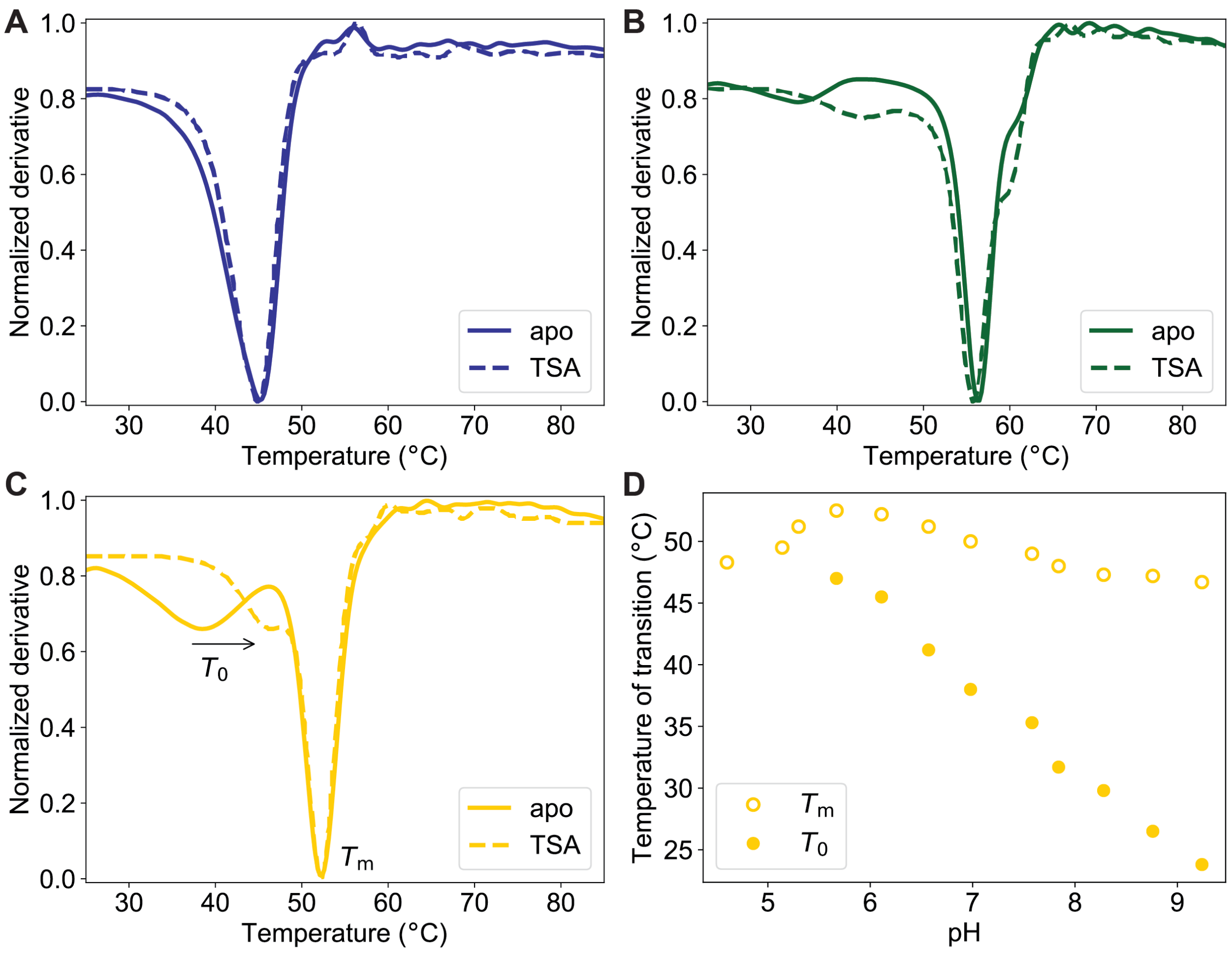

Fig. S3. Melting curves of the HG3 variants reveal conformational sub-states. In contrast to HG3 (**A**), thermal-shift assays of HG3.7 (**B**) and HG3.17 (**C**) show two transitions corresponding to an initial conformational change (T_0) followed by global unfolding (T_m). Addition of the TSA (dashed lines) does not significantly alter T_m, but shifts T_0 toward higher temperature, signaling stabilization of the starting conformational sub-state. In contrast to T_m, T_0 is also pH sensitive and shifts to lower temperatures as pH increases (**D**), which similarly to the effect of TSA addition signals stabilization of the active conformation at lower pH values.

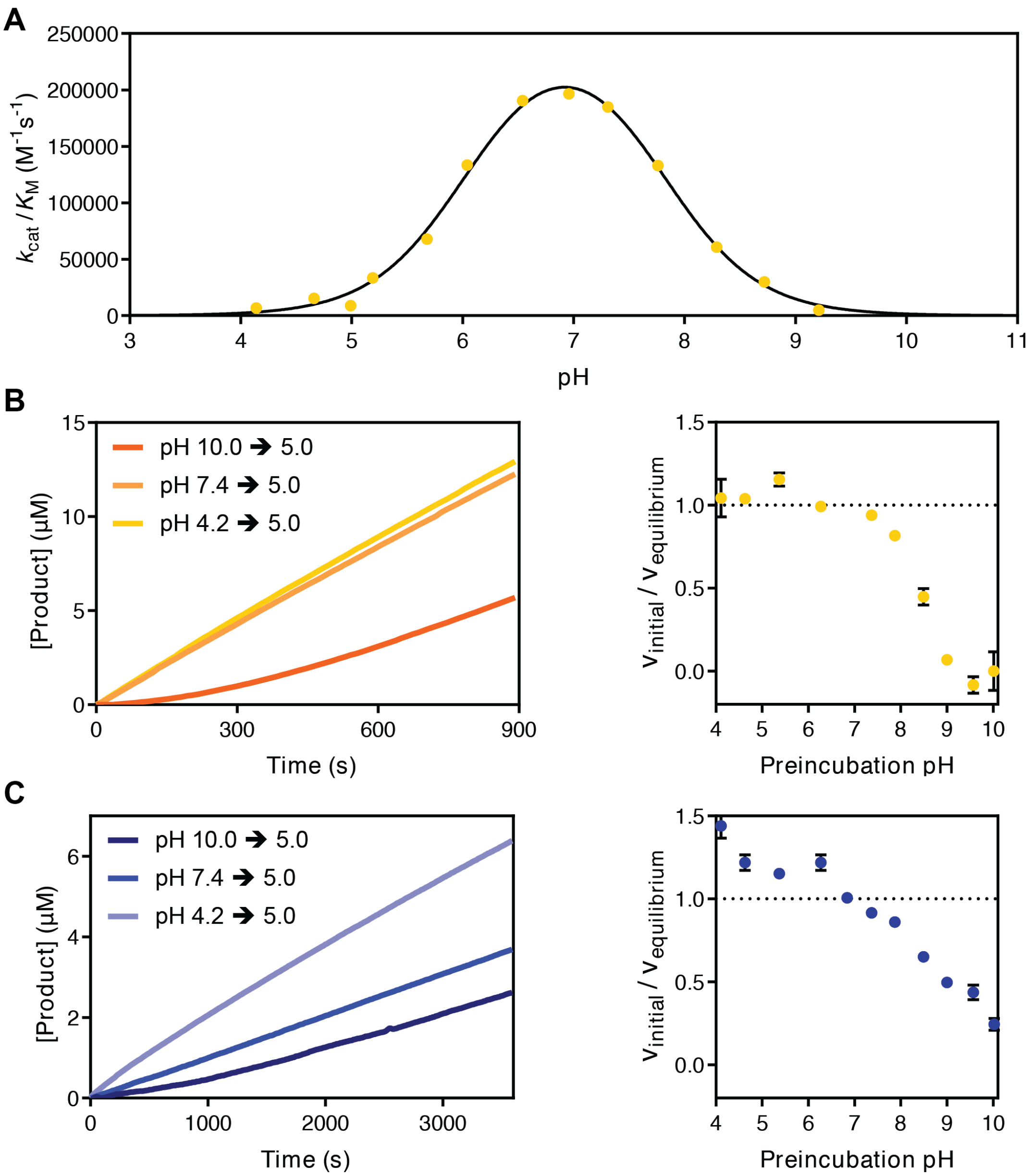

Fig. S4. Slow inactivation of HG3.17 and HG3 at elevated pH measured by enzyme assay. **(A)** HG3.17 has a bell-shaped pH-rate profile. The acidic limb reflects ionization of the catalytic base (pK_a = 6.2), whereas the basic limb reports on the slow and reversible inactivation of the enzyme at elevated pH (pK_a = 7.8). (**B**) pH-jump assays with HG3.17 were performed by incubating the enzyme stock at the indicated pH and then jumping to pH 5 for kinetic measurements at 20 ºC. Enzyme pre-incubated at acidic and neutral pH gives comparable linear initial rates, indicative of similar populations of the active sub-state, whereas enzyme incubated at pH > 8 shows a significant lag phase that increases with increasing pH as expected if conversion of the increasingly populated inactive sub-state back into the active sub-state is slow. (**C**) Analogous pH-jump assays with HG3 show that the active sub-state of the starting design is not fully populated even at pH 7.4.

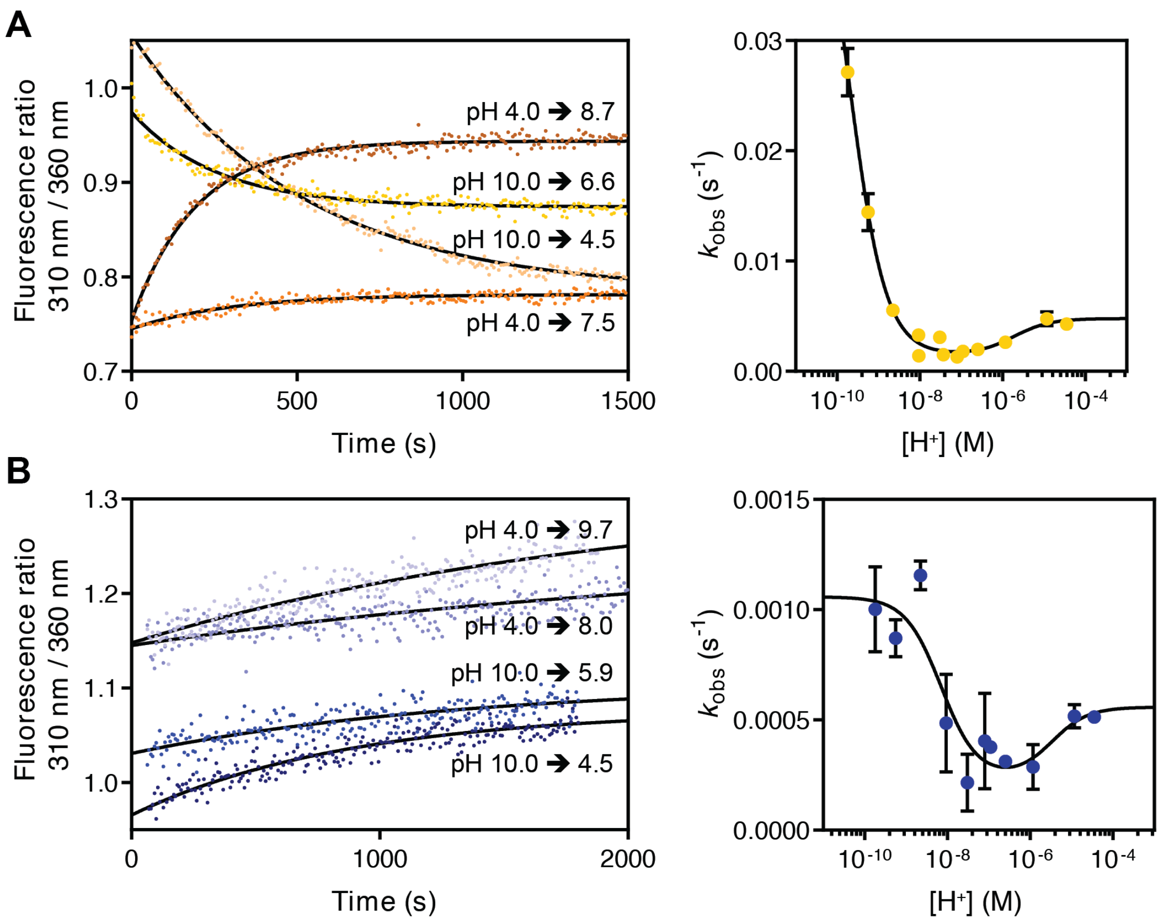

Fig. S5. pH-dependence of the conformational change in HG3 and HG3.17 monitored by tryptophan fluorescence. HG3.17 (**A**) and HG3 (**B**) were incubated either at pH 4.0 or pH 10.0 and the conformational change was monitored by tryptophan fluorescence after jumping to the indicated pH values. The observed equilibration rate constant for the conformational equilibrium has an inverted bell shape, which indicates that the active and inactive states have different pK_a values (*76*). We note that these pK_a values do not reflect (instantaneous) deactivation of the enzyme by protonation of the catalytic base. Instead, deprotonation at another site in the enzyme triggers the (slow) conformational change, which likely modulates the apparent pK_a of the residue in question.

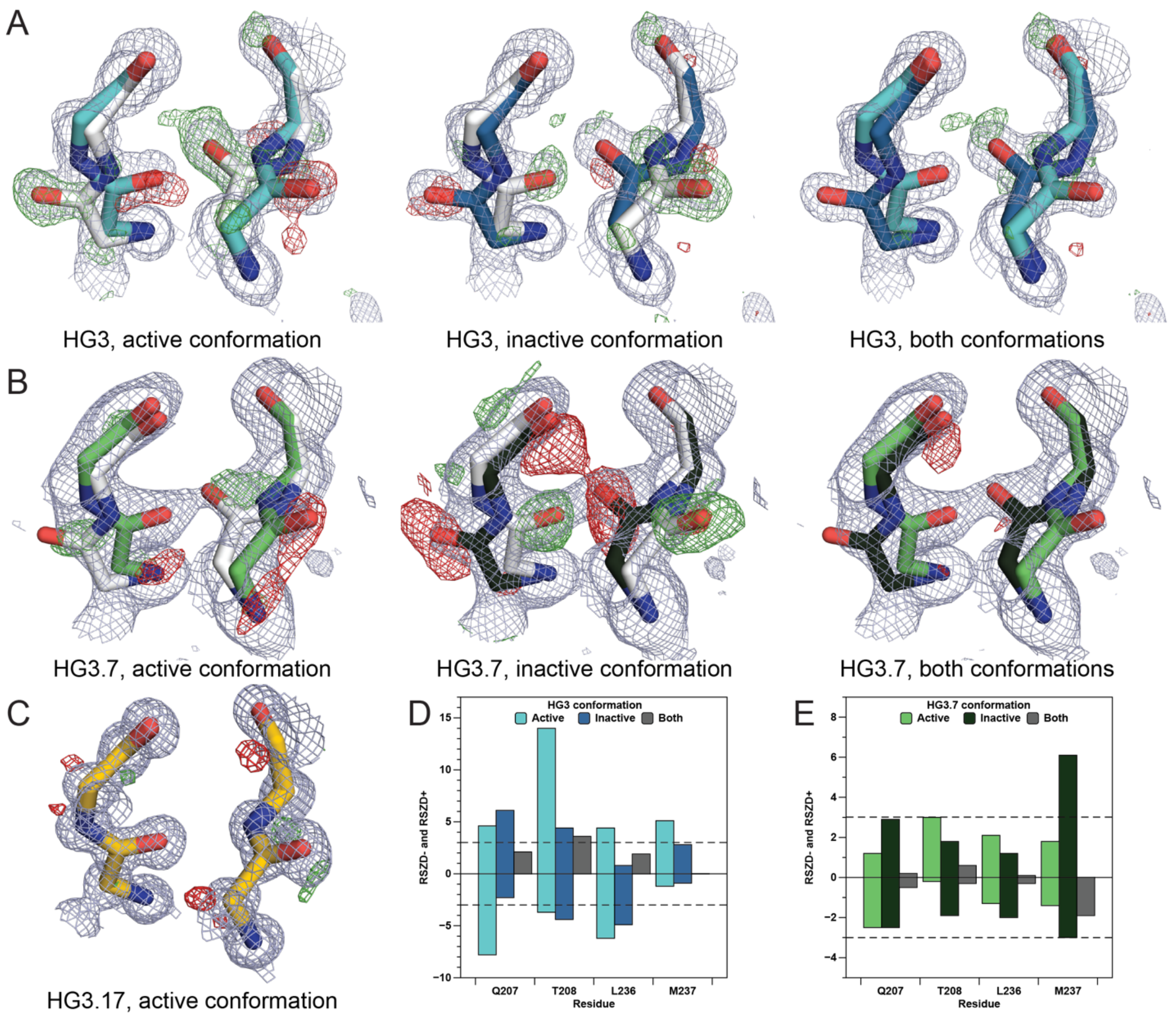

Fig. S6. Difference electron density for alternative conformations of Leu236 and Gln207 in HG3 and HG3.7. Negative and positive difference peaks in mFo-DFc electron density (±3.0σ in green and red, respectively) are contained within 2mFo-DFc electron density (1.0σ in grey). To validate the presence of multiple states refinement cycles were performed setting occupancy to zero for the different states (white models) and occupancy to one for the alternate state. The electron density maps for HG3 (**A**) and HG3.7 (**B**) cannot be accounted for by a single, active or inactive, conformer model. The refined multiconformer models (right) for HG3 (alternative conformations in cyan, 0.49 occupancy, and dark blue, 0.51 occupancy) and HG3.7 (alternative conformations in light green, 0.58 occupancy, and dark green, 0.42 occupancy) explain the difference features. (**C**) In contrast, no difference peaks are observed for HG3.17 when modeling only a single conformation. (**D, E**) Real-space difference density *Z* score (RSZD) (*51*) for both the negative (RSZD-) and positive (RSZD+) density indicates that the local accuracy of the model when fitting both conformations is significantly better than a single, active or inactive, conformer for the residues involved in the backbone flip for HG3 (D) and HG3.7 (E).

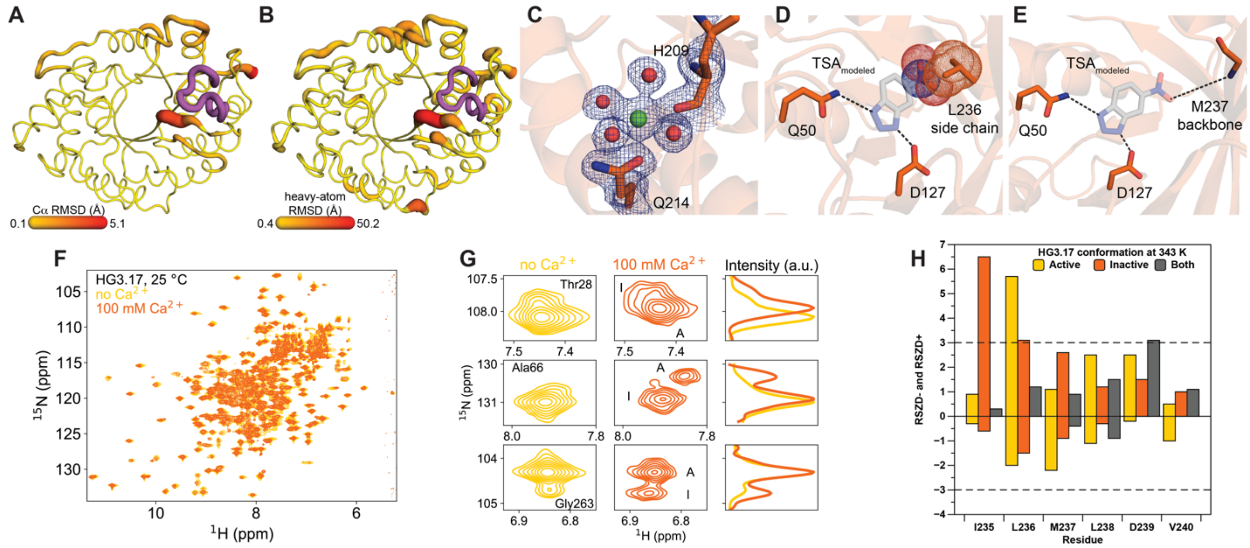

Fig. S7. The inactive conformation of HG3.17 is stabilized by calcium-binding. (**A-B**) RMSD between the active conformation and calcium-bound, inactive conformation of HG3.17 after superpositioning the structures with THESEUS (*49*) highlights the propagation of conformational differences from the active site to remote parts of the protein. RMSD values for Cα atoms (**A**) or the sum of all heavy-atoms (**B**) are shown in sausage representation (with thickness proportional to RMSD value and by the color gradient from yellow to red) onto the active structure. The loop between residues 270-282 that is missing in the inactive conformation is shown in magenta and the RMSD for these residues was set to 2 Å (Cα) or 10 Å (all heavy atoms). (**C**) The calcium ion (green sphere) coordinates with four waters (red spheres), the side-chain oxygen of Gln194, and the amide oxygen of His209. The 2mFo-DFc electron density map is shown as a blue mesh with 1σ contour level. The density was assigned to calcium based on the results of the CheckMyMetal validation server (*52*). The other possibility would be a sodium ion; however, sodium is already present at 150 mM in the buffer of all other structures solved, and we only observe this density when calcium is present in the crystallization condition. (**D**, **E**) The calcium-bound form represents an inactive state because of unfavorable interactions with the TSA. The side chain of Leu236 and the nitro group of the TSA would clash and thus prevent binding (**D**) and the amide nitrogen of Met237 is significantly outside of hydrogen bonding range to the nitro group (**E**, 5.7 Å). (**F, G**) The addition of calcium does not significantly affect the cross-peak positions (chemical shifts) in NMR spectra for HG3.17, indicating that the overall structure of the two conformations is unaltered (**G**). However, calcium-binding increases the population of the inactive conformation (change in intensity) as can be seen in the zoom-ins of the 2D NMR spectrum and their projection onto the ^{15}N axis (**G**). (**H**) Real-space difference density *Z* score (RSZD) (*51*) for both the negative (RSZD-) and positive (RSZD+) density for the data set of HG3.17 measured at high temperature (343 K) as shown in Fig. 3H. These metrics indicate that the local accuracy of the model when fitting both the active and inactive conformations is significantly better than a single conformer for these residues.

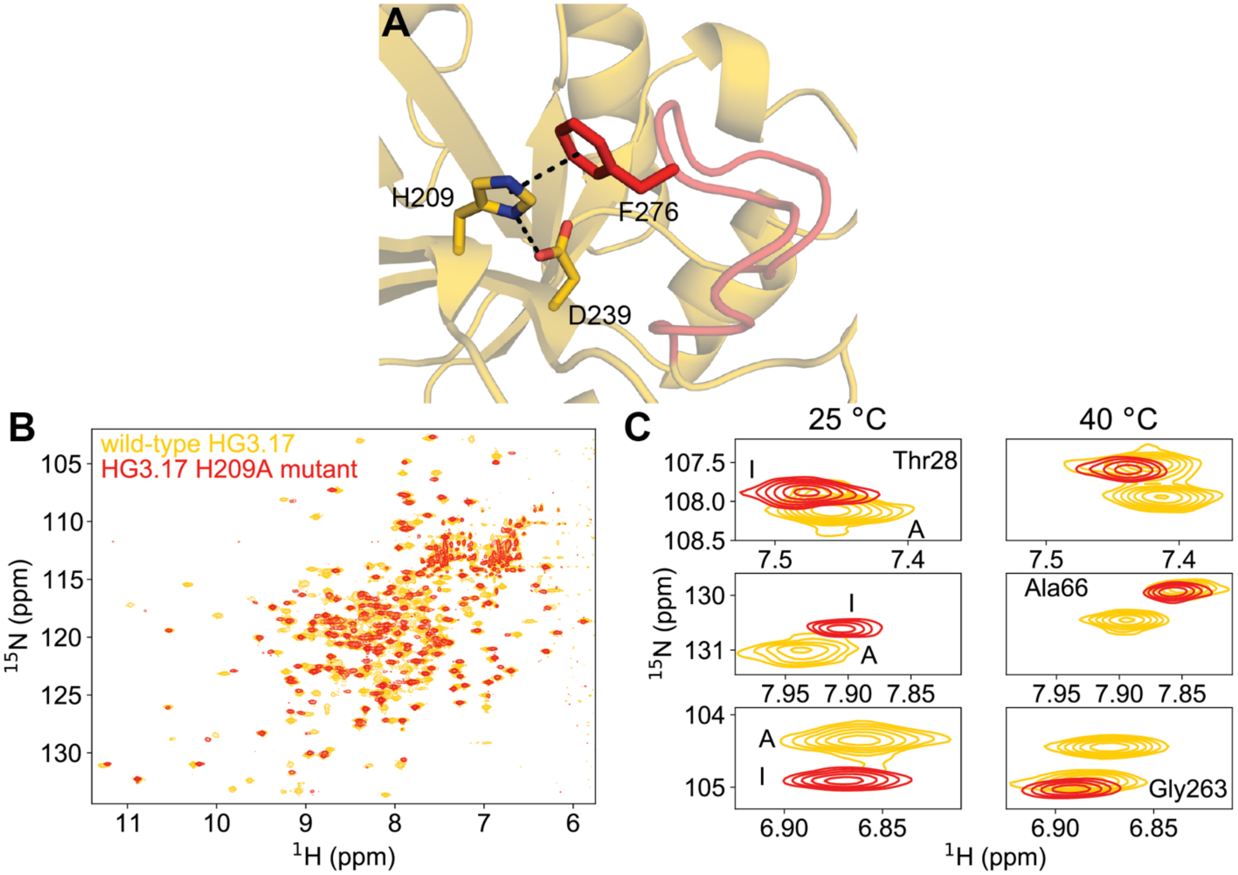

Fig. S8. The possible role of His209 in the active/inactive equilibrium. (**A**) His209 has favorable interactions with Phe276 and Asp239 in the active conformation of HG3.17 as determined using the Arpeggio software (*50*). This interaction appears to order the loop containing Phe276 (residues 270-282, red), which is well resolved in the X-ray structure of HG3.17 shown here. In contrast, there are likely no interactions in the inactive conformation, resulting in more flexibility and the loop being unobservable in the electron density. (**B, C**) 2D [^{1}H, ^{15}N]-TROSY-HSQC spectra of wild-type HG3.17 and its H209A mutant show that the His209 mutant is completely in the inactive conformation already at 25 ºC, suggesting a key role of His209 in the active/inactive equilibrium. Considering the pK_a of the titratable groups in His, we hypothesize that deprotonation of the His209 side chain is likely involved in the inactivation at higher pH.

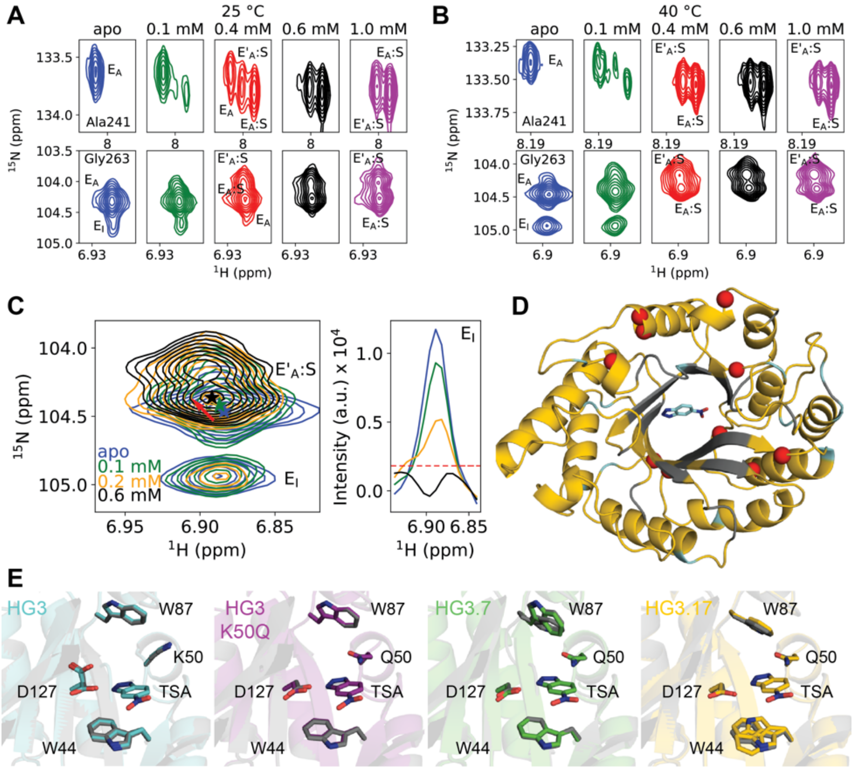

Fig. S9. NMR titration experiment of 500 µM HG3.17 with the transition-state analogue (0 – 1 mM) at 25 ºC and 40 ºC. (**A, B**) Titration data confirm the three-step binding model proposed by the stopped-flow fluorescence experiments: two peaks are observed in the fully saturated TSA-bound form of HG3.17 at both 25 ºC (**A**) and 40 ºC (**B**). The populations of the E_{active}:TSA and E'_{active}:TSA states were determined from the cross-peak volumes of 17 (20) residues at 25 ºC (40 ºC). The calculated equilibrium constants for the induced-fit step are: 1.1 ± 0.9 and 1.3 ± 1.0 at 25 ºC and 40 ºC, respectively. (**C**) The NMR titration patterns strongly suggest that the TSA binds only to the active conformation. Upon addition of the TSA, the cross peak for the active state shifts: the array shows the peak movement upon addition of the TSA, where the peak position is the population weighted average between the cross peaks for E_{active} and E_{active}:TSA. The 'upper' peak at saturating concentrations represents the state after the induced-fit step (E'_{active}:TSA). The 'inactive' cross peak only decreases in intensity and has completely disappeared at higher concentrations (**C**, right panel; red, dotted line represents the noise level). (**D**) Residues that show peak duplication in the TSA-bound form at 25 ºC and/or 40 ºC are shown as red spheres on the structure of HG3.17 with unassigned and Proline residues colored in gray and cyan, respectively. (**E**) Only for HG3.17 an induced-fit step is observed and we speculate that a Trp residue in the binding pocket might be involved. X-ray structures of Kemp variants (shown in color and gray for the TSA-bound and free forms, respectively) show that Trp87 has the same conformation for all variants irrespective of the presence of TSA. In contrast, Trp44 in HG3.17 is present in two distinct orientations: one of them is also observed in the free form, whereas the other is exclusively seen in the TSA-bound state. For all other variants the Trp44 side chain is in a single conformation and does not change upon TSA binding. Such a ring flip in the crowded interior of the β-barrel is likely slow, long-range aromatic effects could explain the peak duplication, and the change in environment would explain the signal observed in Trp-fluorescence stopped-flow experiments.

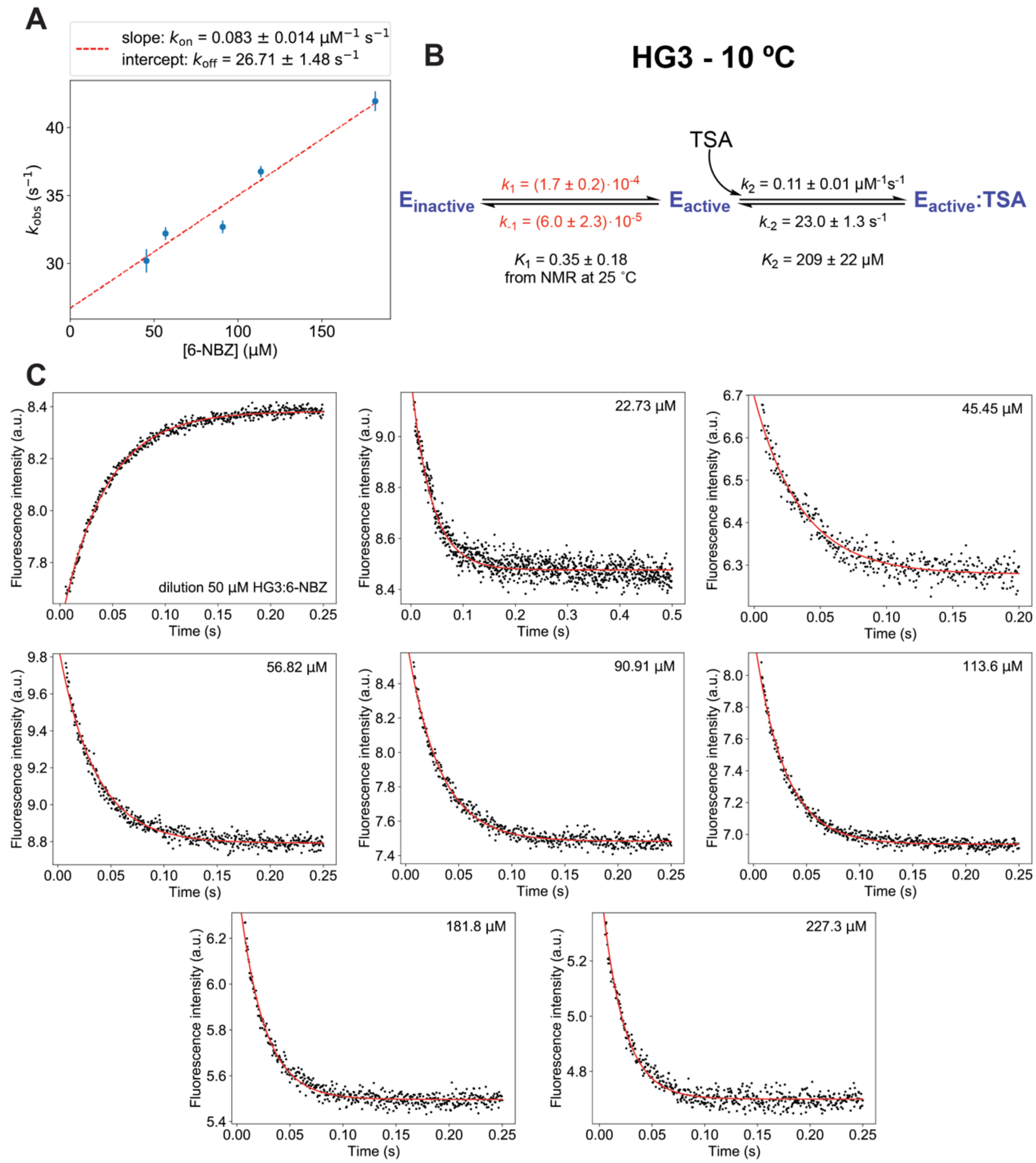

Fig. S10. Binding kinetics of TSA binding to HG3 measured by stopped-flow Trp fluorescence experiments at 10 °C. (**A**) Observed rate constants plotted against TSA concentration show a linear dependency, representing the binding step. (**B**) Transition-state analog (labeled as TSA) binding scheme to HG3 corresponds to a two-step binding mechanism: conformational selection followed by the physical binding step; the corresponding microscopic rate constants shown are obtained from the global fit (**C**; χ^2/DoF = 1.03). The interconversion between $E_{inactive}$ and E_{active} is too slow to measure using stopped-flow fluorescence and the microscopic rate constants for this step are derived from NMR experiments (see Figs. 2 and 4A). Fluorescence traces are the average of at least five replicate measurements (n > 5), error bars in panel **A** are derived from fitting the individual time traces, and uncertainties given in the scheme denote the (propagated) standard deviation in the fitted parameters.

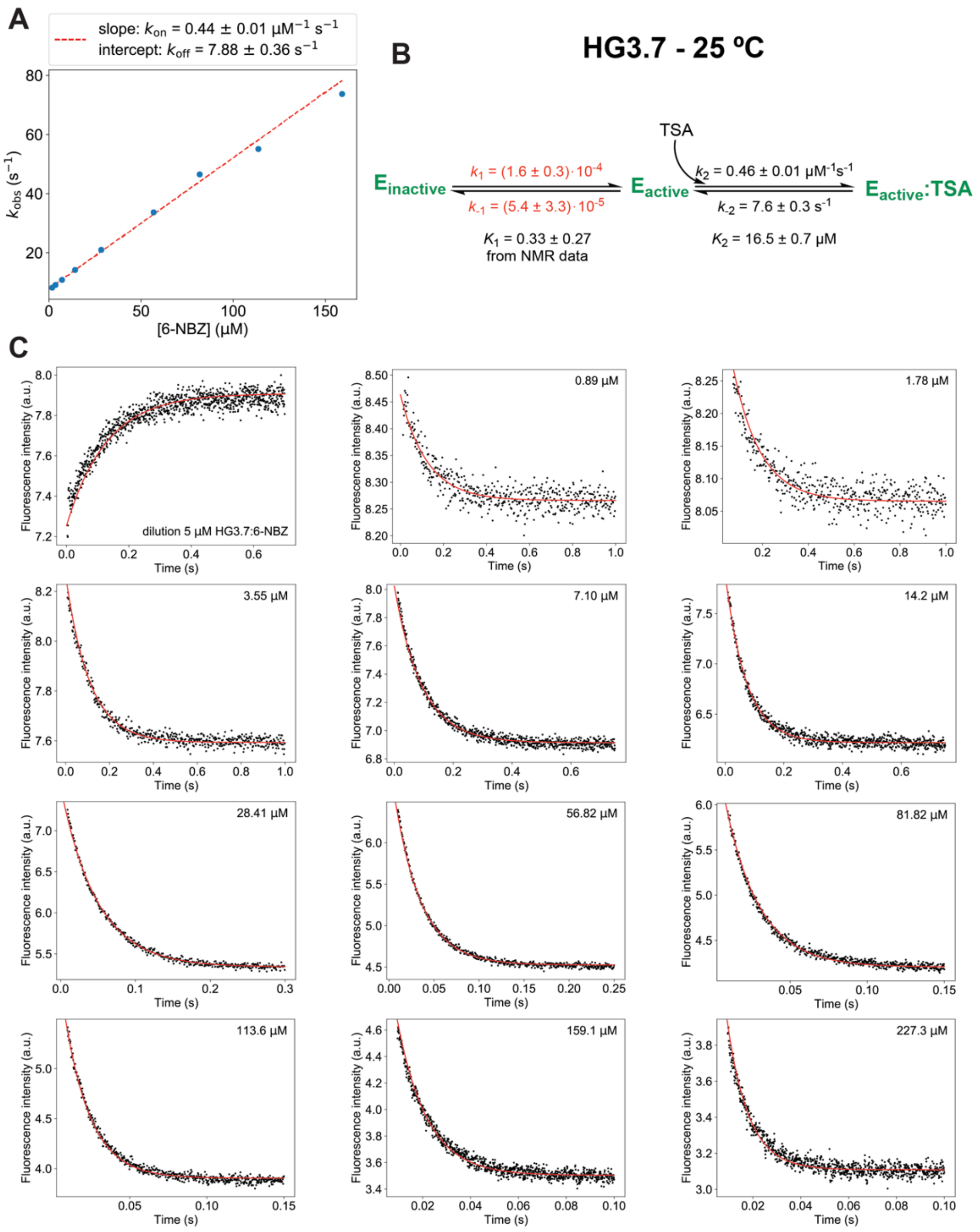

Fig. S11. Binding kinetics of TSA binding to HG3.7 measured by stopped-flow Trp fluorescence experiments at 25 °C. (**A**) Observed rate constants plotted against TSA concentration show a linear dependency, representing the binding step. (**B**) Transition-state analog (labeled as TSA) binding scheme to HG3.7 corresponds to a two-step binding mechanism: conformational selection followed by the physical binding step; the corresponding microscopic rate constants shown are obtained from the global fit (**C**; χ^2/DoF = 1.17). The interconversion between $E_{inactive}$ and E_{active} is too slow to measure using stopped-flow fluorescence and the microscopic rate constants for this step are derived from NMR experiments (see Figs. 2 and 4A). Fluorescence traces are the average of at least five replicate measurements (n > 5), error bars in panel A are derived from fitting the individual time traces, and uncertainties given in the scheme denote the (propagated) standard deviation in the fitted parameters.

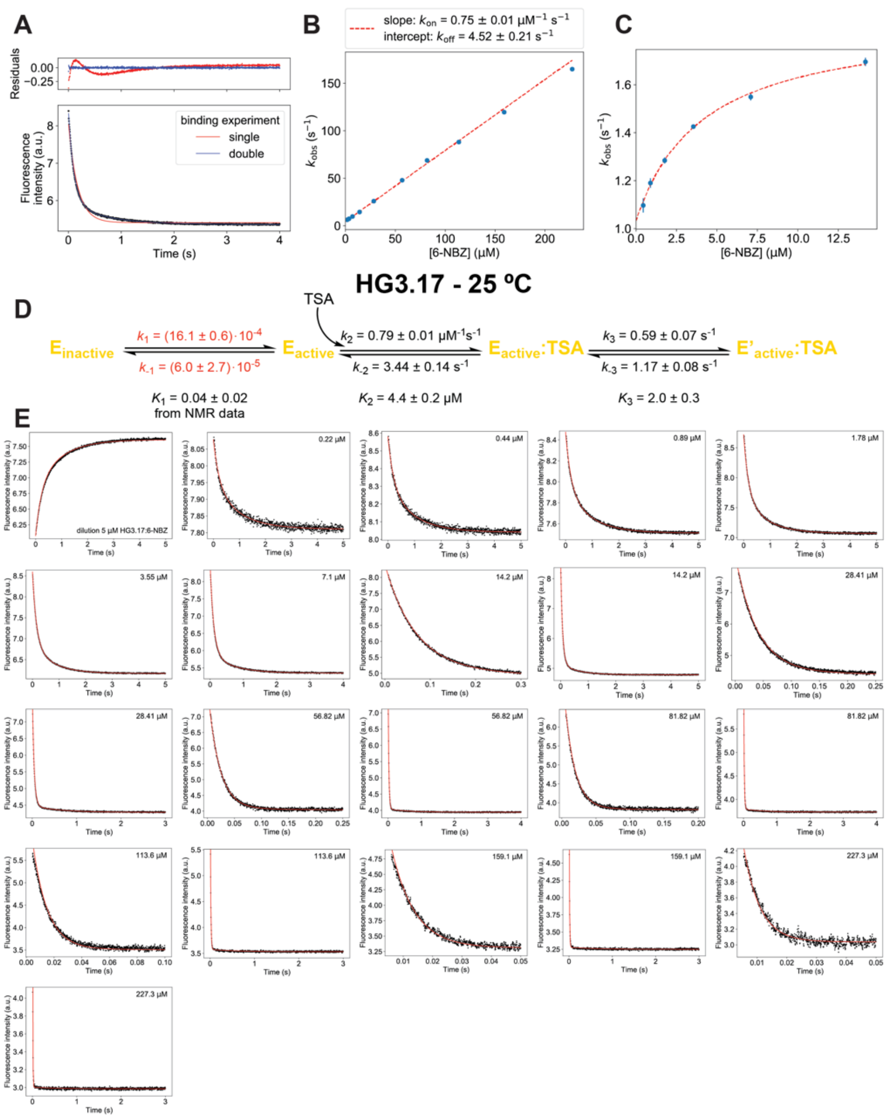

Fig. S12. Binding kinetics of TSA binding to HG3.17 measured by stopped-flow Trp fluorescence experiments at 25 ºC. (**A**) Comparison of fitting the fluorescence time traces to a single (red) and double (blue) exponential function show that this is a three-step binding mechanism. (**B-C**) Observed rate constants plotted against TSA concentration show a linear dependency for the fast phase, indicating the binding step (**A**); the slow phase shows a non-linear dependency and indicates a protein conformational transition (**B**). (**C**) Transition-state analog (labeled as TSA) binding scheme to HG3.17 corresponds to a three-step binding mechanism: conformational selection followed by the physical binding step and induced-fit step; the corresponding microscopic rate constants shown are obtained from the global fit (**D**; χ^2/DoF = 1.76). The interconversion between $E_{inactive}$ and E_{active} is too slow to measure using stopped-flow fluorescence and the microscopic rate constants for this step are derived from NMR experiments (see Figs. 2 and 4A). Fluorescence traces are the average of at least five replicate measurements (n > 5), error bars in panels **B** and **C** are derived from fitting the individual time traces, and uncertainties given in the scheme denote the (propagated) standard deviation in the fitted parameters.

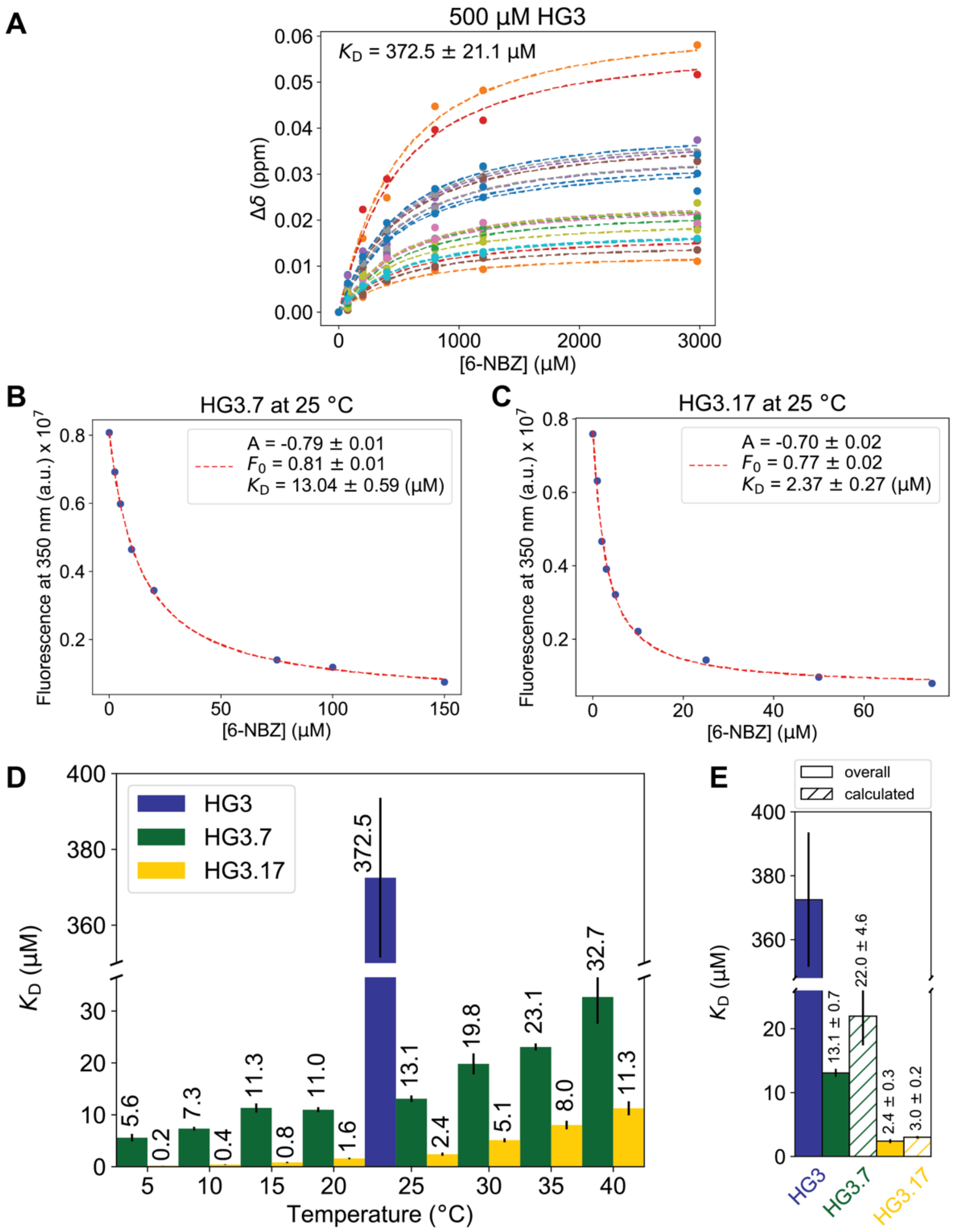

Fig. S13. Macroscopic dissociation constants (K_D) between the TSA and HG3 variants. (**A**) The overall K_D for the TSA and HG3 at 25 ºC was determined by NMR titration due to significant inner-filter effects in a fluorescence experiment at the high TSA concentrations required to saturate HG3. Twenty-one resonances with a sufficient signal-to-noise ratio and $\Delta\delta \geq 0.015$ ppm (Equation 1) were simultaneously fit to Equation 2 and the standard error is obtained from the global fit. (**B-D**) Trp fluorescence titration experiments were used to measure the overall K_D for the TSA and HG3.7 (**B**, at 25 ºC) and HG3.17 (**C**, at 25 ºC) at different temperatures (**D**). (**E**) The overall macroscopic K_D values for Kemp eliminase variants to the TSA agree with the values calculated from the microscopic rate constants using Equations 11 and 12, respectively.

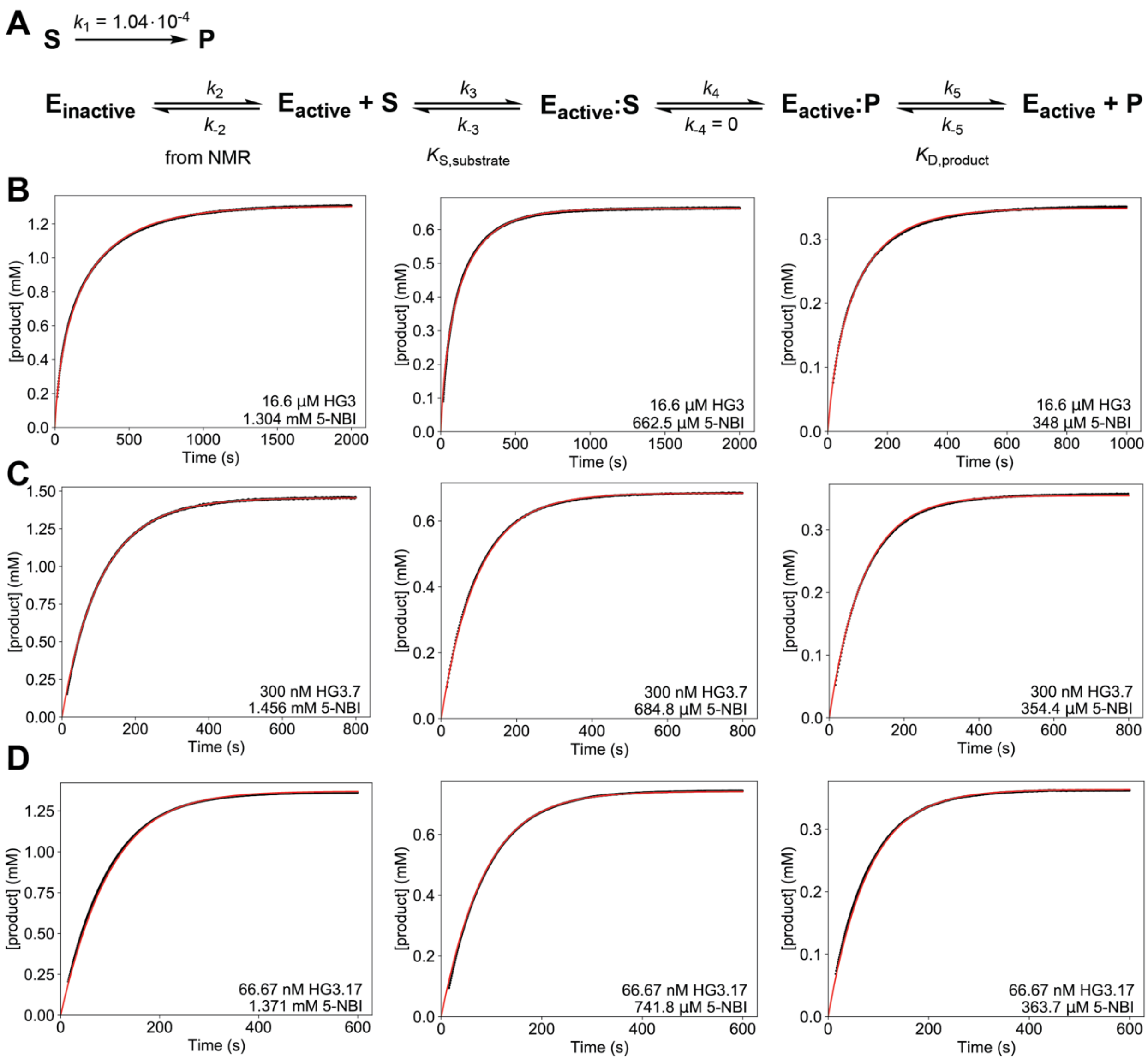

Fig. S14. Progress curves of product formation provide more accurate steady-state parameters for HG3, HG3.7, and HG3.17. (**A**) The complete kinetic scheme used for fitting the progress curves includes the spontaneous background reaction from substrate (5-NBI, 5-nitrobenzisoxazole) to product and the catalyzed reaction by HG3 (**B**), HG3.7 (**C**), and HG3.17 (**D**). Fitting was performed as described in the Materials and Methods section and the steady-state parameters for k_{cat}, $K_{M,substrate}$, $K_{S,substrate}$, and $K_{D,product}$ are calculated according to Equations 3-6 and listed in Fig. 4B. The fitted $K_{D,product}$ values for HG3 and HG3.17 agree qualitatively with those determined from preliminary NMR titration experiments (13.2 ± 0.8 mM; HG3.17) and ITC data (~0.22 mM; HG3).

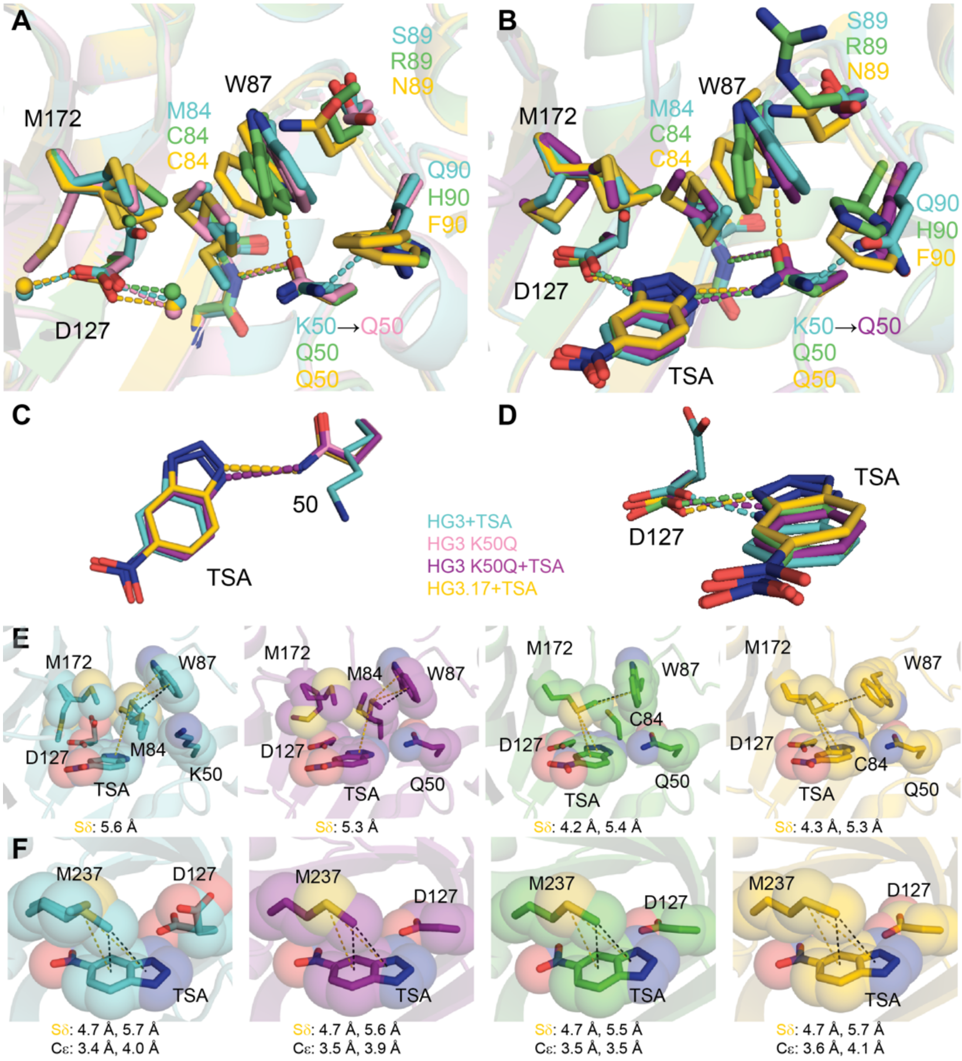

Fig. S15. X-ray crystallography data provide a rationale for improved catalysis in evolved variants. (**A**, **B**) Overlay of active-site residues for Kemp eliminase variants in the free (**A**) or TSA-bound (**B-D**) state with their favorable interactions as determined by Arpeggio (*50*). A comparison of the two panels indicates that TSA binding does not alter the side-chain conformations of Asp127. Asp127 hydrogen bonds with water molecules in the free state and with the triazole ring in the TSA-bound form. Second- and third-shell mutations ultimately result in an active site with positioning of the oxyanion stabilizer (Gln50) and catalytic base (Asp127) perfectly oriented for catalysis. In HG3, Lys50 originally designed as oxyanion stabilizer cannot serve in this function due to interactions with Gln90. Note an additional interaction of Gln50 with Trp87 in HG3.17. (**C, D**) Zoom-in of TSA-interactions with residue 50 and Asp127 for structures shown in (A, B). (**E**) Favorable (dispersive) interactions between the TSA and Met172 can only occur after the M84C mutation in HG3.7. In HG3 and HG3 K50Q, Met84 interacts with the triazole ring and Trp87. (**F**) Favorable (dispersive) interactions between the TSA and Met237 are present in all variants. Distances listed are from the sulfur atom (Sδ) or methyl group (Cε) to the center of mass of the benzyl- and/or triazole-ring of the TSA.

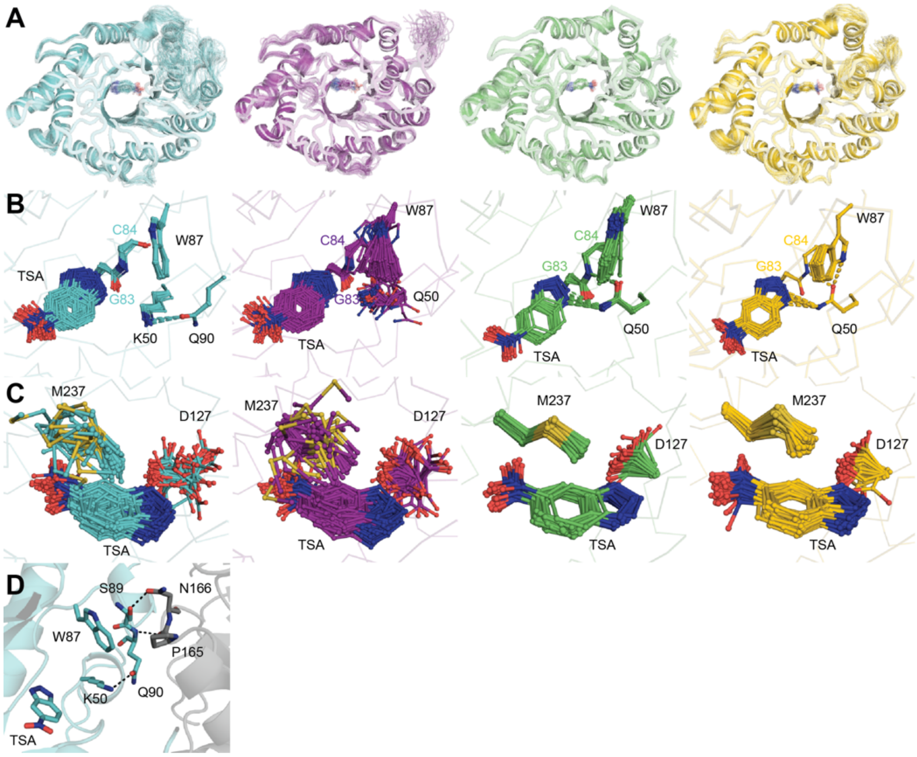

Fig. S16. Ensemble refinement of the TSA-bound forms of HG3, HG3 K50Q, HG3.7, and HG3.17 illustrates gradual decrease of sampling of unreactive conformations of active-site residues during directed evolution. All data sets were recorded at cryogenic temperature and refinements were performed using phenix.ensemble_refinement (*18*) as described in the Materials and Methods with the default value for wxray (wxray_coupled_tbath_offset = 5.0) and the optimal values for parameters pTLS and tx determined automatically. The maximum resolution in the ensemble refinement was limited to 1.8 Å (1.77 Å for HG3.17) to minimize the contribution of radiation damage which is greater at higher resolution shells. Overall view (**A**) and corresponding zoom-ins (**B**-**C**) for wild-type HG3 (cyan, 75 conformers; refined with pTLS 0.8 and tx 0.6), HG3 K50Q (purple, 67 conformers; refined with pTLS 0.8 and tx 0.6), HG3.7 (green, 34 conformers; refined with pTLS 0.9 and tx 0.6), and HG3.17 (yellow, 67 conformers; refined with pTLS 0.9 and tx 0.6). (**B, C**) Extensive side chain heterogeneity is observed for wild-type HG3 and HG3 K50Q, whereas in evolved enzymes side chains orientations become progressively more ordered and are primed for catalysis. The apparent order for residues Lys50 and Trp87 in HG3 is explained by crystal contacts in that region that are specific to HG3 (**D**). HG3 K50Q is thus better suited for comparison of the ensembles as it forms similar crystal contacts as HG3.7 and HG3.17**.** (**B**) Lys50 in HG3 does not interact with the TSA, but instead hydrogen-bonds to the side chain of Gln90. The K50Q mutation introduces the oxyanion hole stabilizing group, but there is still significant heterogeneity that precludes a stable interaction with the TSA. In HG3.7 and HG3.17 the side chain of Gln50 is in a single conformation with favorable interactions to the TSA and stabilized by an additional hydrogen bond to Gly83. Due to a change in rotameric state of Trp87 in HG3.17, an additional hydrogen bond is formed between Gln50 and Trp87 that potentially further stabilizes Gln50 in HG3.17. Residue labels shown in color indicate that backbone atoms are shown, whereas in all other cases the sticks represent the side chains. (**C**) The side chain of Met237 becomes more ordered through evolution, which allows for additional favorable interactions with the TSA.

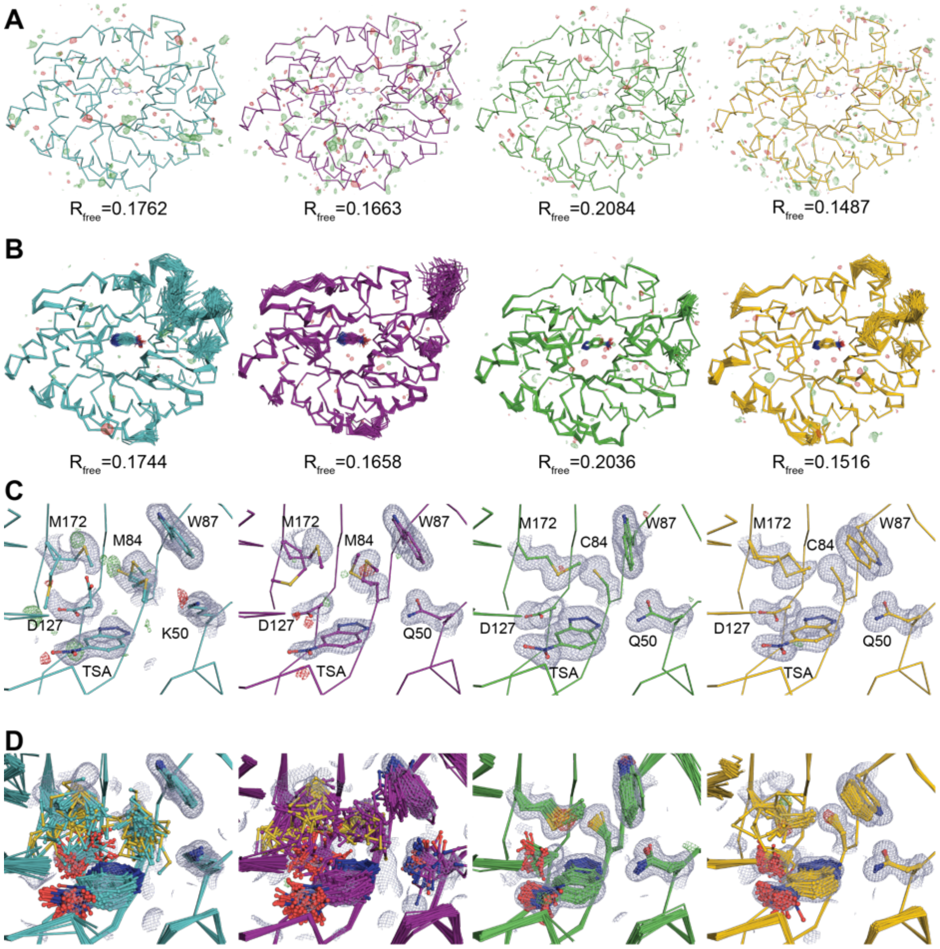

Fig. S17. Ensemble refinement of the TSA-bound forms of HG3, HG3 K50Q, HG3.7, and HG3.17 shows better agreement with the electron density map compared to single structure refinement. All data sets were recorded at cryogenic temperature and refinements were performed as described in the legend of Fig. S16. **(A)** The density difference maps (mFo-DFc) for the single structures refined with phenix.refine are shown contoured at 3.00 σ level with the following absolute map levels (HG3, 0.42 e/Å^3, cyan; HG3 K50Q, 0.38 e/Å^3, purple; HG.7, 0.34 e/Å^3, green; HG3.17, 0.45 e/Å^3, yellow). The majority of the peaks in the difference map in **(A)** are resolved when fitting an ensemble instead of a single structure to the crystallography data **(B)** (the absolute map levels in (A) were used as a reference and the displayed contour levels are: HG3 3.53 σ, HG3 K50Q 3.71 σ, HG3.7 3.36 σ, HG3.17 3.65 σ). The overall lowering in R_{free} indicates that including more states does not cause overfitting. The slight increase in R_{free} between the single structure and ensemble refinements for HG3.17 is not fully understood and is under investigation by the software developers. **(C)** 2mFo-DFc maps (grey) at 1.00σ level show that the electron density for important active site residues and the TSA becomes more well defined in the fastest variants which results in more ordered ensembles for the evolved kemp eliminases **(D)**.

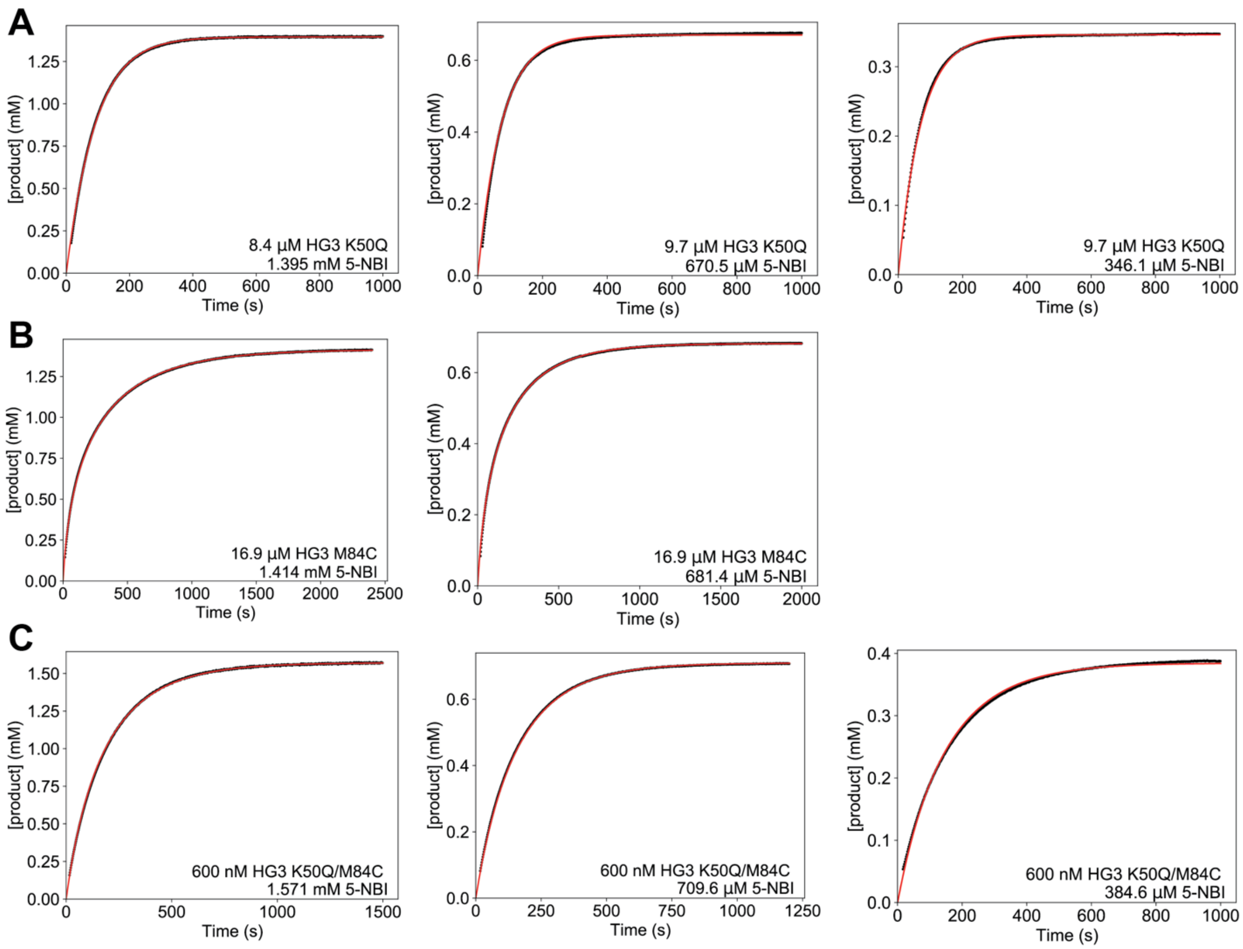

D

HG3	K50Q	M84C	K50Q/M84C
k_{cat} (s^{-1})	12.2 ± 1.0	10.2 ± 4.1	230 ± 79
$K_M^{substrate}$ (mM)	8.2 ± 0.8	13.4 ± 5.8	19.3 ± 6.8
k_{cat} / K_M (M^{-1} s^{-1})	(1.5 ± 0.2) x 10^3	(0.8 ± 0.4) x 10^3	(1.2 ± 0.6) x 10^4
$K_D^{product}$ (mM)	17.0 ± 4.6	0.24 ± 0.01	2.0 ± 0.1

Fig. S18. Progress curves of product formation to determine the steady-state parameters for single- and double-mutants of HG3. Fitting to the scheme in Fig. S14A was performed as described in the Materials and Methods section and is shown for HG3 K50Q (**A**), HG3 M84C (**B**), and HG3 K50Q/M84C (**C**). It was assumed that the values for k_2 and k_{-2} are the same as for wild-type HG3. The steady-state parameters for k_{cat}, $K_{M,substrate}$, and $K_{D,product}$ are calculated according to Equations 3-6 and are shown in the table (**D**).

HG3:
MAEAAQSVDQ LIKARGKVYF GVATDQNRLT TGKNAAIIQA DFGMVWPENS MKWDATEPSQ
GNFNFAGADY LVNWAQQNGK LIGGGMLVWH SQLPSWVSSI TDKNTLTNVM KNHITTLMTR
YKGKIRAWDV VGEAFNEDGS LRQTVFLNVI GEDYIPIAFQ TARAADPNAK LYIMDYNLDS
ASYPKTQAIV NRVKQWRAAG VPIDGIGSQT HLSAGQGAGV LQALPLLASA GTPEVSILML
DVAGASPTDY VNVVNACLNV QSCVGITVFG VADPDSWRAS TTPLLFDGNF NPKPAYNAIV
QDLQQ

HG3 K50Q:
MAEAAQSVDQ LIKARGKVYF GVATDQNRLT TGKNAAIIQA DFGMVWPENS MQWDATEPSQ
GNFNFAGADY LVNWAQQNGK LIGGGMLVWH SQLPSWVSSI TDKNTLTNVM KNHITTLMTR
YKGKIRAWDV VGEAFNEDGS LRQTVFLNVI GEDYIPIAFQ TARAADPNAK LYIMDYNLDS
ASYPKTQAIV NRVKQWRAAG VPIDGIGSQT HLSAGQGAGV LQALPLLASA GTPEVSILML
DVAGASPTDY VNVVNACLNV QSCVGITVFG VADPDSWRAS TTPLLFDGNF NPKPAYNAIV
QDLQQ

HG3 M84C:
MAEAAQSVDQ LIKARGKVYF GVATDQNRLT TGKNAAIIQA DFGMVWPENS MKWDATEPSQ
GNFNFAGADY LVNWAQQNGK LIGGGCLVWH SQLPSWVSSI TDKNTLTNVM KNHITTLMTR
YKGKIRAWDV VGEAFNEDGS LRQTVFLNVI GEDYIPIAFQ TARAADPNAK LYIMDYNLDS
ASYPKTQAIV NRVKQWRAAG VPIDGIGSQT HLSAGQGAGV LQALPLLASA GTPEVSILML
DVAGASPTDY VNVVNACLNV QSCVGITVFG VADPDSWRAS TTPLLFDGNF NPKPAYNAIV
QDLQQ

HG3 K50Q/M84C:
MAEAAQSVDQ LIKARGKVYF GVATDQNRLT TGKNAAIIQA DFGMVWPENS MQWDATEPSQ
GNFNFAGADY LVNWAQQNGK LIGGGCLVWH SQLPSWVSSI TDKNTLTNVM KNHITTLMTR
YKGKIRAWDV VGEAFNEDGS LRQTVFLNVI GEDYIPIAFQ TARAADPNAK LYIMDYNLDS
ASYPKTQAIV NRVKQWRAAG VPIDGIGSQT HLSAGQGAGV LQALPLLASA GTPEVSILML
DVAGASPTDY VNVVNACLNV QSCVGITVFG VADPDSWRAS TTPLLFDGNF NPKPAYNAIV
QDLQQ

HG3.7: V6I, Q37K, K50Q, M84C, S89R, Q90H, A125N
MAEAAQSIDQ LIKARGKVYF GVATDQNRLT TGKNAAIIKA DFGMVWPENS MQWDATEPSQ
GNFNFAGADY LVNWAQQNGK LIGGGCLVWH RHLPSWVSSI TDKNTLTNVM KNHITTLMTR
YKGKIRNWDV VGEAFNEDGS LRQTVFLNVI GEDYIPIAFQ TARAADPNAK LYIMDYNLDS
ASYPKTQAIV NRVKQWRAAG VPIDGIGSQT HLSAGQGAGV LQALPLLASA GTPEVSILML
DVAGASPTDY VNVVNACLNV QSCVGITVFG VADPDSWRAS TTPLLFDGNF NPKPAYNAIV
QDLQQ

HG3.17: V6I, Q37K, N47E, K50Q, G82A, M84C, S89N, Q90F, T105I, A125T, T142N, T208M, F267M, W275A, R276F, T279S, D300N
MAEAAQSIDQ LIKARGKVYF GVATDQNRLT TGKNAAIIKA DFGMVWPEES MQWDATEPSQ
GNFNFAGADY LVNWAQQNGK LIGAGCLVWH NFLPSWVSSI TDKNTLINVM KNHITTLMTR
YKGKIRTWDV VGEAFNEDGS LRQNVFLNVI GEDYIPIAFQ TARAADPNAK LYIMDYNLDS
ASYPKTQAIV NRVKQWRAAG VPIDGIGSQM HLSAGQGAGV LQALPLLASA GTPEVSILML
DVAGASPTDY VNVVNACLNV QSCVGITVMG VADPDSAFAS STPLLFDGNF NPKPAYNAIV
QNLQQ

Fig. S19. Amino acid sequences of HG3 variants. Amino acid changes between HG3 with its point mutants and the evolved enzymes, HG3.7 and HG3.17, are color-coded on the sequence. Of note, the purification-tag used leaves an additional GS at the N-terminus that is now shown. The sequence numbering used in the manuscript follows that of the original HG2 variant (*7*). where the first two residues were not observable in the X-ray structure and, therefore, starts with residue number 1 for E at position 3 in the listed sequence.

	HG3	HG3 + TSA	HG3 K50Q	HG3 K50Q + TSA	HG3.7	HG3.7 + TSA	HG3.17	HG3.17 + Calcium	HG3.17 E47N N300D	HG3.17 E47N N300D + TSA	HG3.17 E47N N300D 343 K
PDB	7k4p	7k4q	7k4r	7k4u	7k4s	7k4x	7k4v	7k4w	7k4t	7k4z	7k4y
Data collection [a]											
Beamline	14-1 SSRL	14-1 SSRL	821 ALS	821 ALS	821 ALS	821 ALS	821 ALS	501 ALS	821 ALS	821 ALS	7-1 SSRL
Wavelength (Å)	0.9537	1.1271	1.000	1.000	0.9998	0.8855	0.9999	0.9774	0.8000	0.8000	0.9795
Resolution range (Å)	32.93 - 1.081 (1.12 - 1.081)	48.59 - 1.277 (1.323 - 1.277)	37.85 - 1.57 (1.626 - 1.57)	33.39 - 1.3 (1.346 - 1.3)	41.55 - 1.8 (1.864 - 1.8)	41.11 - 1.6 (1.657 - 1.6)	48.39 - 1.3 (1.346 - 1.3)	50.04 - 1.9 (1.968 - 1.9)	40.11 - 0.999 (1.035 - 0.999)	35.84 - 0.999 (1.035 - 0.999)	36.86 - 1.8 (1.864 - 1.8)
Space group	$P2_1$	$P2_1$	$P2_12_12_1$	$P2_12_12_1$	$P2_12_12_1$	$P2_12_12_1$	$P2_12_12_1$	$P2_12_12_1$	$P2_12_12_1$	$P2_12_12_1$	$P2_12_12_1$
Unit cell	50.61 58.03 81.61 90 101.42 90	50.64 58.97 88.76 90 104.94 90	48.258 61.002 81.632 90 90 90	48.089 59.829 80.468 90 90 90	50.47 68.22 73.21 90 90 90	75.17 77.33 97.09 90 90 90	49.19 65.67 71.57 90 90 90	61.39 67.39 74.7 90 90 90	50.15 66.82 71.98 90 90 90	49.36 66.31 71.68 90 90 90	49.71 68.12 73.72 90 90 90
Total reflections	1186260 (113894)	805475 (81074)	242406 (24095)	397528 (35242)	129263 (12864)	150112 (14634)	374629 (31045)	298305 (28654)	872475 (65634)	806023 (62165)	126276 (12671)
Unique reflections	188303 (18272)	126111 (12643)	34365 (3369)	57674 (5663)	24041 (2351)	75249 (7438)	57517 (5622)	25033 (2465)	131238 (12932)	127672 (12643)	23826 (2330)
Multiplicity	6.3 (6.2)	6.4 (6.4)	7.1 (7.2)	6.9 (6.2)	5.4 (5.5)	2.0 (2.0)	6.5 (5.5)	11.9 (11.6)	6.6 (5.1)	6.3 (4.9)	5.3 (5.4)
Completeness (%)	95.18 (92.71)	96.27 (97.15)	99.96 (100.00)	99.57 (97.97)	97.85 (85.58)	99.96 (99.91)	99.39 (97.38)	99.82 (99.15)	99.95 (99.94)	97.22 (74.03)	99.85 (99.87)
Mean I/sigma(I)	6.37 (1.70)	10.07 (2.99)	7.82 (1.06)	8.33 (0.60)	3.52 (0.32)	5.93 (0.74)	9.80 (0.94)	4.74 (0.79)	11.30 (1.73)	5.94 (0.29)	7.38 (0.99)
Wilson B-factor ($Å^2$)	9.76	11.43	13.72	13.28	20.94	17.94	16	20.6	6.79	8.82	22.15
R_{merge} [b]	0.1196 (1.015)	0.0722 (0.5134)	0.2554 (2.828)	0.1761 (3.621)	0.4621 (4.347)	0.059 (0.7947)	0.08381 (2.239)	0.5383 (3.51)	0.09251 (0.8555)	0.1506 (4.256)	0.1564 (1.81)
$CC_{1/2}$ [c]	0.998 (0.548)	0.999 (0.919)	0.995 (0.322)	0.995 (0.222)	0.959 (0.0142)	0.997 (0.428)	0.998 (0.319)	0.979 (0.251)	0.998 (0.631)	0.997 (0.0668)	0.987 (0.286)
Refinement											
Reflections used in refinement	187977 (18263)	125995 (12628)	34363 (3369)	57538 (5549)	23542 (2012)	75233 (7432)	57356 (5530)	25011 (2450)	131202 (12932)	124197 (9381)	23815 (2327)
Reflections used for R_{free}	1997 (194)	1989 (202)	2000 (196)	1995 (193)	1959 (166)	2003 (199)	1990 (187)	1999 (197)	2009 (198)	1937 (131)	2023 (208)
R_{work} [d]	0.1788 (0.3746)	0.1671 (0.2746)	0.1681 (0.3013)	0.1852 (0.3866)	0.2113 (0.4222)	0.1931 (0.3841)	0.1592 (0.4024)	0.2165 (0.3773)	0.1590 (0.2587)	0.1554 (0.4173)	0.1619 (0.3498)
R_{free} [e]	0.2076 (0.3494)	0.1865 (0.2944)	0.2008 (0.3228)	0.2114 (0.3768)	0.2639 (0.4545)	0.2249 (0.4018)	0.1765 (0.4324)	0.2525 (0.3663)	0.1813 (0.2882)	0.1740 (0.4277)	0.2005 (0.3656)
Number of non-hydrogen atoms	6939	5741	2891	2798	2758	5314	2699	2461	3069	2750	4554
macromolecules	6319	4908	2508	2507	2439	4713	2417	2300	2624	2431	4475
ligands	7	34	-	12	20	70	13	1	-	28	-
solvent	613	799	383	279	299	531	269	160	445	291	79
Protein residues	609	602	305	306	301	607	300	289	301	302	300
RMSD (bonds)	0.013	0.004	0.007	0.006	0.002	0.005	0.016	0.003	0.009	0.011	0.006
RMSD (angles)	1.28	0.79	0.79	0.9	0.46	0.76	1.39	0.55	1.09	1.21	0.93

Ramachandran favored (%)	97.66	97.82	98.33	98.68	97.32	98	98.65	98.25	99	98.66	97.32
Ramachandran allowed (%)	2.34	2.18	1.67	1.32	2.68	2	1.35	1.75	1	1.34	2.68
Ramachandran outliers (%)	0	0	0	0	0	0	0	0	0	0	0
Rotamer outliers (%)	1.06	0.75	1.85	1.12	1.15	0.2	0	0	0.71	1.16	2.53
Clashscore	3.75	3.17	3.81	4.61	1.85	3.6	2.89	1.76	1.53	2.05	3.83
Average B-factor	13.83	15.31	17.8	18.99	26.02	24.99	20.88	27.33	8.5	12.65	24.78
macromolecules	12.81	13.36	15.69	17.33	24.56	23.85	19.6	27.05	6.97	11.22	24.66
ligands	37.24	22.21	-	27.74	56.14	44.77	40.75	63.74	-	19.4	22.3
solvent	24.08	26.99	31.65	33.45	35.91	32.5	31.45	31.09	17.53	23.94	31.22

[a] Values in parentheses correspond to the data in the highest resolution shell

[b] $R_{merge} = \frac{\sum_i \sum_{j=1}^{n_i} \left| I_j(hkl) - \overline{I(hkl)} \right|}{\sum_i \sum_{j=1}^{n_i} I_j(hkl)}$, where $I_j(hkl)$ is the intensity of an unique reflection and $\overline{I(hkl)}$ is the average of multiple reflections

[c] $CC_{1/2}$ is the Pearson's correlation coefficient between intensities from two half-datasets

[d] $R_{work} = \frac{\sum_{hkl} \left| |F_{obs}(hkl)| - |F_{calc}(hkl)| \right|}{\sum_{hkl} |F_{obs}(hkl)|}$, where $F_{obs}(hkl)$ is the observed structure factor and $F_{calc}(hkl)$ is the structure factor calculated from the model

[e] R_{free} is calculated using the R_{work} equation but with a randomly selected set of reflections that is excluded from refinement

Table S1. X-ray crystallography data collection and refinement statistics.

Kemp variant	Codon-optimized DNA sequence
HG3	AGCGGCATGGCGGAGGCGGCGCAGAGCGTGGACCAACTGATTAAGGCGCGTGGCAAGGTGTATTTC<br>GGCGTGGCGACCGACCAGAACCGTCTGACCACCGGCAAGAACGCGGCGATCATTCAGGCGGACTTC<br>GGCATGGTGTGGCCGGAGAACAGCATGAAATGGGATGCGACCGAACCGAGCCAGGGTAACTTCAA<br>CTTTGCGGGCGCGGACTACCTGGTTAACTGGGCGCAGCAAAACGGCAAGCTGATCGGTGGCGGTAT<br>GCTGGTGTGGCACAGCCAGCTGCCGAGCTGGGTTAGCAGCATTACCGATAAGAACACCCTGACCAA<br>CGTGATGAAAAACCACATCACCACCCTGATGACCCGTTATAAGGGTAAAATTCGTGCGTGGGACGT<br>GGTTGGCGAGGCGTTCAACGAAGATGGCAGCCTGCGTCAGACCGTGTTTCTGAACGTTATCGGCGA<br>GGACTACATCCCGATTGCGTTTCAGACCGCGCGTGCGGCGGACCCGAACGCGAAACTGTACATCAT<br>GGACTATAACCTGGATAGCGCGAGCTATCCGAAGACCCAGGCGATTGTGAACCGTGTTAAACAATG<br>GCGTGCGGCGGGTGTGCCGATTGATGGTATTGGTAGCCAGACCCATCTGAGCGCGGGTCAGGGTGC<br>GGGCGTTCTGCAAGCGCTGCCGCTGCTGGCGAGCGCGGGCACCCCGGAAGTGAGCATTCTGATGCT<br>GGATGTTGCGGGTGCGAGCCCGACCGATTACGTTAACGTGGTTAACGCGTGCCTGAACGTGCAAAG<br>CTGCGTTGGTATTACCGTGTTTGGTGTTGCGGACCCGGATAGCTGGCGTGCGAGCACCACCCCGCTG<br>CTGTTTGATGGTAACTTTAACCCGAAACCGGCGTATAATGCGATTGTTCAGGATCTGCAACAGTAA
HG3.7	CATATGGCGGGCAGCCACCACCACCACCACCACGGTATGGCGAGCATGACCGGCGGCCAACAAATG<br>GGTCGTAGCGGCGATGACGATGACAAGGAGAACCTGTACTTTCAAGGCAGCGGTATGGCGGAAGCG<br>GCGCAGAGCATCGACCAACTGATTAAGGCGCGTGGTAAAGTGTATTTCGGCGTTGCGACCGATCAA<br>AACCGTCTGACCACCGGCAAGAACGCGGCGATCATTAAAGCGGACTTTGGCATGGTGTGGCCGGAG<br>AACAGCATGCAGTGGGATGCGACCGAACCGAGCCAGGGTAACTTCAACTTTGCGGGCGCGGACTAC<br>CTGGTTAACTGGGCGCAGCAAAACGGTAAACTGATCGGTGGCGGTTGCCTGGTGTGGCACCGTCAC<br>CTGCCGAGCTGGGTTAGCAGCATTACCGATAAGAACACCCTGACCAACGTGATGAAAAACCACATC<br>ACCACCCTGATGACCCGTTATAAGGGTAAAATTCGTAACTGGGACGTGGTTGGCGAGGCGTTCAAC<br>GAAGATGGCAGCCTGCGTCAGACCGTGTTTCTGAACGTTATCGGCGAGGACTACATCCCGATTGCGT<br>TCCAAACCGCGCGTGCGGCGGACCCGAACGCGAAGCTGTACATCATGGACTATAACCTGGATAGCG<br>CGAGCTATCCGAAGACCCAGGCGATTGTGAACCGTGTTAAACAATGGCGTGCGGCGGGTGTGCCGA<br>TTGATGGTATTGGTAGCCAGACCCATCTGAGCGCGGGTCAGGGTGCGGGCGTTCTGCAGGCGCTGC<br>CGCTGCTGGCGAGCGCGGGTACCCCGGAAGTGAGCATTCTGATGCTGGATGTTGCGGGTGCGAGCC<br>CGACCGATTACGTTAACGTGGTTAACGCGTGCCTGAACGTGCAGAGCTGCGTTGGTATTACCGTGTT<br>TGGTGTTGCGGACCCGGATAGCTGGCGTGCGAGCACCACCCCGCTGCTGTTTGACGGCAACTTTAAC<br>CCGAAACCGGCGTATAATGCGATTGTTCAAGACCTGCAACAGTAAGGATCC
HG3.17	CATATGGCGGGTTCTCATCATCATCATCATCATGGTATGGCTAGCATGACTGGTGGCCAGCAAATGG<br>GTCGCTCCGGTGATGATGATGACAAGGAAAACTTGTACTTTCAAGGCTCAGGTATGGCAGAAGCGG<br>CTCAATCCATTGACCAACTGATTAAAGCTCGTGGTAAAGTGTATTTCGGTGTTGCAACCGACCAGAA<br>CCGCCTGACAACTGGCAAGAACGCGGCGATCATTAAGGCAGATTTCGGTATGGTTTGGCCAGAGGA<br>GTCCATGCAATGGGACGCGACCGAGCCTTCTCAAGGCAACTTCAACTTCGCTGGCGCAGACTACCTG<br>GTGAACTGGGCGCAGCAGAACGGTAAGCTGATCGGCGCTGGTTGTCTGGTCTGGCACAATTTTCTGC<br>CGTCTTGGGTGTCTTCCATCACAGATAAAAACACTCTGATCAACGTGATGAAGAACCATATCACTAC<br>CCTGATGACCCGTTATAAAGGCAAAATCCGCACCTGGGATGTTGTGGGCGAAGCATTCAACGAGGA<br>CGGCAGCCTGCGCCAGAACGTTTTTCTGAATGTGATCGGTGAAGATTACATCCCGATCGCATTCCAG<br>ACCGCCCGTGCTGCAGATCCAAACGCTAAGCTGTACATTATGGATTACAACCTGGACTCTGCGTCTT<br>ATCCGAAAACCCAGGCCATCGTGAACCGTGTTAAACAGTGGCGTGCGGCTGGCGTTCCGATTGACG<br>GCATCGGCTCCCAGATGCATCTGTCAGCAGGCCAGGGCGCTGGCGTGCTGCAGGCCCTGCCGCTGCT<br>GGCAAGCGCCGGCACTCCGGAGGTTAGCATCTTGATGCTGGATGTAGCGGGCGCAAGCCCGACTGA<br>TTATGTGAACGTCGTGAACGCATGTCTGAATGTGCAGTCTTGTGTGGGCATTACCGTAATGGGTGTT<br>GCCGATCCTGATTCTGCGTTTGCGTCCAGTACCCCGCTGCTGTTCGATGGTAATTTCAACCCGAAAC<br>CAGCTTACAACGCTATCGTTCAGAACTTGCAACAGTAAGGATCC

Table S2. Codon-optimized DNA sequence for Kemp eliminase variants.

Variant	Forward primer	Reverse primer
HG3 K50Q	5'– GCC GGA GAA CAG CAT GCA GTG GGA TGC GAC CGA –3'	3'– GTT CGG TCG CAT CCC ACT GCA TGC TGT TCT CCG GC –5'
HG3 M84C	5'– CTG ATC GGT GGC GGT TGC CTG GTG TGG CAC AGC –3'	3'– GCT GTG CCA CAC CAG GCA ACC GCC ACC GAT CAG –5'
HG3.17 H209A	5'– TCG GCT CCC AGA TGG CTC TGT CAG CAG GCC –3'	3'– GGC CTG CTG ACA GAG CCA TCT GGG AGC CGA –5'
sequencing primer	5'– GGG TGT CTT CCA TCA CAG ATA AAA ACA CTC TGA TCA ACG –3'	3'– CGT TGA TCA GAG TGT TTT TAT CTG TGA TGG AAG ACA CCC –5'

Table S3. Primer sequences used for generating the HG3 and HG3.17 mutants and internal sequencing primer.
